# Characterization of GH18 chitinase in *Leishmania braziliensis*: expression, structural insights, and implications for vaccine and therapeutic development

**DOI:** 10.1186/s40659-026-00685-y

**Published:** 2026-04-16

**Authors:** Maura Rojas-Pirela, Marirene Chacón-Arnaude, Cristóbal Lárez-Velásquez, María Verónica Rojas, Constanza Cardenas, Ana J. Cáceres, Luis-Antonio Corchete, Diego Andrade-Alviárez, Luis A. Prieto-Rojas, Daniel Salete-Granado, María Á. Pérez-Nieto, Wilfredo Quiñones, Paul A. M. Michels, Miguel Marcos

**Affiliations:** 1 Instituto de Investigación Biomédica de Salamanca (IBSAL), 37007 Salamanca, Spain; 2 Departamento de Medicina, Universidad de Salamanca, 37007 Salamanca, Spain; 3 Servicio de Medicina Interna, Hospital Universitario de Salamanca, 37007 Salamanca, Spain; 4 Laboratorio de Enzimología de Parásitos, Departamento de Biología, Facultad de Ciencias, Universidad de Los Andes, Mérida, 5101 Venezuela; 5 Max Planck Institute for Evolutionary Biology, Plön, Germany; 6 Grupo de Polímeros, Departamento de Química, Universidad de Los Andes, Mérida, Venezuela; 7 Instituto de Biología, Facultad de Ciencias, Pontificia Universidad Católica de Valparaíso, 2373223 Valparaíso, Chile; 8 Núcleo de Biotecnología Curauma, Pontificia Universidad Católica de Valparaíso, Av. Universidad 330. 2373223, Valparaíso, Chile; 9 Krantz Family Center for Cancer Research, Massachusetts General Hospital, Charlestown, MA USA; 10 Harvard Medical School, Boston, MA USA; 11 Broad Institute of MIT and Harvard, Cambridge, MA USA; 12 Facultad de Medicina, Universidad de Los Andes, Mérida, 5101 Venezuela; 13 School of Biological Sciences, University of Edinburgh, The King’s Buildings, Edinburgh, EH9 3FL UK; 14 Faculty of Applied Sciences, UCSI University, Kuala Lumpur, Malaysia

**Keywords:** *L. braziliensis*, GH18 chitinase, Kinetoplastid evolution, Therapeutic targets, vaccine candidates

## Abstract

Chitinases, a group of glycosyl hydrolases (GHs), catalyze the degradation of chitin by releasing N-acetylglucosamine subunits. GHs can be found in all three domains of life. Among the three GH families (GH18, GH19, and GH20), GH18 chitinases are the most conserved and extensively studied. These enzymes have been implicated in nutrition, immune modulation, invasion, and virulence across diverse pathogens. In some parasitic protists, GH18 chitinases are essential for transmission. However, in kinetoplastids, including *Leishmania* spp., these enzymes remain poorly characterized. This study aimed to identify and characterize chitinase across kinetoplastids, with a particular focus on GH18 chitinase in *Leishmania braziliensis*, the causative agent of muco-cutaneous leishmaniasis. A bioinformatic pipeline was implemented to retrieve and annotate chitinase genes from multiple databases. Catalytic domains and subcellular localization were identified using dedicated computational tools. Phylogenetic relationships were reconstructed in MEGA12 using maximum likelihood and neighbor-joining methods. RNA-seq data were analyzed to evaluate stage-specific expression. Structural models of *L. braziliensis* GH18 chitinase (Lbr_ChGH18) were created with AlphaFold and subsequently refined. We identified GH18 chitinases genes across *Leishmania* species and other kinetoplastids, together with GH19 and GH20 chitinase genes. Lbr_ChGH18 was detected across all life-cycle stages, with peak levels in amastigotes. Docking analyses identified closantel, argifin, and argadin as potential inhibitors of Lbr_ChGH18. Epitope prediction revealed conserved B- and T-cell epitopes in GH18 chitinases from Old and New World *Leishmania*, capable of binding multiple HLA class I and II molecules, highlighting their potential as diagnostic and vaccine candidates. The discovery of GH20 chitinase-like genes in *Trypanosoma* species offers new insights into the evolution and functional diversification of GHs in kinetoplastids. The high conservation and expression of GH18 chitinases underscore their potential as promising targets for drug and vaccine development against leishmaniasis.

**Supplementary Information:**

The online version contains supplementary material available at 10.1186/s40659-026-00685-y.

## Introduction

Chitin is a linear β-1,4-linked homopolymer of N-acetyl-D-glucosamine (GlcNAc), one of the most ancient and abundant structural polysaccharides in nature [1, 2]. Traditionally associated with fungi, crustaceans, and arthropods, chitin has also been detected in vertebrates, including humans [3], which can produce short chitin oligomers—precursors of glycosaminoglycan disaccharides such as those in hyaluronic acid, chondroitin sulfate, and keratan sulfate [4]. These findings have renewed interest in identifying genes encoding chitinases and chitin synthase-type enzymes involved in GlcNAc chain assembly and remodeling.

Chitinases are glycosyl hydrolases (GH) that catalyze the degradation of chitin by releasing GlcNAc moieties. They are classified into three families, GH18, 19, and 20, which differ in their amino-acid sequences and catalytic properties [5, 6]. GH18 and GH19 are enzymes that catalyze the hydrolysis of β-1, 4-N-acetyl-D-glucosamine linkages in chitin polymers, while family GH20 members (which includes chitobiases and β-N-acetylhexosaminidases) catalyze the hydrolysis of dimeric units of N-acetylglucosamine (chitobiose) as well as terminal N-acetylgalactosamine or glucosamine from glycoconjugates [5, 6]. Among these families, GH18 is the most studied and widespread across the three domains of life. Structurally, GH18 chitinases are characterized by a catalytic region that consists of a so-called TIM barrel (β/α)_8_ domain and a conserved DXXDXDXE or DXDXE sequence motif within this barrel, essential for enzymatic activity [5, 7]. GH18 enzymes are multifunctional, participating in chitin degradation and remodeling, immune system modulation, invasion, and pathogenesis [8]. They enhance virulence in pathogenic bacteria and viruses by facilitating host invasion and nutrient acquisition, while modulating host immune responses [9].

In parasites, this chitinase remained so far relatively understudied; however, in the apicomplexan protist *Plasmodium* and the nematode *Brugia*, GH18 chitinases act as stage-specific molecules crucial for transmission, highlighting their potential value as targets for transmission-blocking and vaccine strategies [10, 11]. In parasitic kinetoplastid protists, chitinases remain poorly characterized. These enzymes have been explored in some *Leishmania* species [12–16], causative agents of leishmaniasis, a neglected tropical disease (NTD) affecting globally over 12 million people. This disease is endemic in 90 countries and presents in different clinical manifestations, classified into three forms: cutaneous leishmaniasis (CL), mucosal/mucocutaneous leishmaniasis (ML/MCL), and visceral leishmaniasis (VL). ML can cause disfiguring damage, social stigma, and even death, whereas CL typically leaves lifelong scars [17]. VL, in contrast, is a systemic disease and primarily affects vulnerable populations, including children under five years of age, adults over 50, and individuals with comorbidities or immunosuppression. Without timely treatment, the case fatality rate exceeds 90% [17].

GH18 chitinases are highly conserved across *Leishmania*, suggesting critical roles in parasite biology [12, 15]. In *L. mexicana* and *L. donovani,* GH18 chitinases have been linked to parasite development in the insect vector and enhance transmission to the vertebrate host [14, 16, 18]. They have been proposed as multifunctional virulence factors [14] and potential antigens [19], although their specific epitopes have not yet been characterized. Notably, this chitinase is highly expressed in amastigotes, which enhances parasite survival in macrophages and lesion severity in murine models [14], highlighting it as a key virulence determinant and a potential target for therapeutic and vaccine development. Despite these advances, studies addressing the structural characteristics of GH18 chitinases in *Leishmania* remain very scarce.

Conversely, in other kinetoplastids, such as *Trypanosoma brucei*, chitinases have been reported inconsistently [20, 21], while they were not detected in other *Trypanosoma* species [12, 15].

Current therapeutic options for leishmaniasis are limited by toxicity, cost, resistance, and reduced efficacy, and the lack of effective vaccines underscores the urgent need for new therapeutic targets [22]. Clinical management remains heterogeneous and context-dependent, making species identification crucial for optimizing treatment and improving outcomes [23]. Importantly, leishmaniasis also affects domestic animals, particularly dogs, which can serve as reservoirs for human infection [24]. Although current treatments can reduce parasite load and clinical signs, none ensures complete parasite clearance, highlighting the persistent risk of transmission [25]. In this context, exploring parasite-specific proteins and enzymes represents a promising avenue for identifying novel therapeutic and diagnostic targets.

Over 20 *Leishmania* species infect humans, with *L. braziliensis*, a New World species, causing cutaneous and mucocutaneous leishmaniasis. This species represents a suitable research model due to its well-annotated genomic and transcriptomic resources [26]. Therefore, this study aimed to provide the first integrative characterization of GH18 chitinases in *L. braziliensis* (Lbr_ChGH18), combining comparative genomics, transcriptomics, structure modeling, and immunoinformatic analyses.

Examining the genome sequences, we identified a single GH18 chitinase gene in several pathogenic *Leishmania* species, most of them predicted to be catalytically active and either to be secreted or membrane associated. In Lbr_ChGH18, expression was detected across all life-cycle stages, with the highest levels observed in amastigotes. This study constitutes the first report of structural analyses of a GH18 chitinase in *Leishmania,* providing novel insights into its molecular architecture. Protein structural and compound-docking analyses revealed potential inhibitory interactions with closantel, argifin, and argadin, well-known chitinase inhibitors, suggesting opportunities for drug repurposing or structure-based inhibitor design, with potential applications in the treatment of leishmaniasis in humans and animal hosts. Epitope prediction revealed conserved antigenic regions across GH18 chitinases, supporting their relevance as vaccine and diagnostic candidates. Moreover, GH20 chitinase-like genes were detected in *Trypanosoma* species, an observation not previously reported, which opens new opportunities for comparative and cross-species studies.

## Methods

### Identification and bioinformatic characterization of chitinase genes in kinetoplastids

A comprehensive analysis of chitinase genes in kinetoplastids and other protists was conducted through a systematic approach to identify, annotate, and characterize their sequences. Chitinase gene sequences were retrieved from the TriTryp [27], InterPro [28], Ensembl Protists [29], and National Center for Biotechnology Information (NCBI) [30] databases. The presence and verification of catalytic domains within these sequences were assessed using multiple bioinformatics tools and servers, including Protein BLAST (NCBI), the Conserved Domain Database (CDD) [31], InterProScan (EMBL-EBI) [32], and the Simple Modular Architecture Research Tool (SMART) [33], with e-values ranging from 3.06e^−242^ to 1.71e^−11^, ensuring high-confidence domain identification. The subcellular localization of the identified chitinases was determined based on literature-reported data and predictions obtained from specialized computational tools, namely WoLF PSORT [34], DeepLoc [35], and Cell-Ploc [36].

### Multiple sequence alignment and phylogenetic analysis of chitinases

Sequences of chitinases were retrieved from the TriTryp [27], InterPro [28], and the NCBI databases and uploaded in FASTA format. Multiple sequence alignments were generated with Clustal Omega (EMBL-EBI) [37] and MUSCLE (EMBL-EBI) [38], and manually curated in MEGA12 [39]. A phylogenetic tree was constructed with 42 chitinase amino-acid sequences from three distinct families (33 GH18, 5 GH19, and 4 GH20) using the Neighbor-Joining method [40]. A bootstrap test (1000 replicates) was performed to determine the percentage of replicate trees in which the associated taxa clustered together [41]. The evolutionary distances were computed using the Poisson correction method [42], and expressed in the units of the number of amino-acid substitutions per site. All positions containing alignment gaps and missing data were eliminated only in pairwise sequence comparisons (Pairwise deletion option). The sequences used in the analysis are listed, each with its TriTrypDB, GeneDB, or NCBI accession code, in Supplementary Methodology. There was a total of 526 positions in the final dataset. Phylogenetic analyses were performed in MEGA12 [39].

### RNA-seq data compilation and expression profiling of GH18 chitinases in *L. braziliensis*

Given the limited information on GH18 chitinase expression across *L. braziliensis* developmental stages, RNA-seq data from publicly available repositories were analyzed to characterize their transcriptional profiles. Data from the RNA-seq library (SRP162992) were retrieved from the TriTryp database [27] and Ruy et al., 2019 [43], to assess gene expression across parasite developmental stages, including amastigote, promastigote, and metacyclic forms. A detailed overview of the dataset, including sequencing platform, read counts per library, and biological replicates, is provided in Supplementary Methodology. Differential expression analyses were performed using DESeq2 v1.46.0 [44]. Raw read counts were normalized using the median-of-ratios method (size factor normalization) implemented in DESeq2 to account for differences in sequencing depth among libraries. Differential expressions across developmental stages was determined using the Wald test with Benjamini–Hochberg correction (adjusted *p*-value < 0.05). For visualization purposes, log₂-transformed normalized counts were used.

### Structure modeling and docking analyses of the *L. braziliensis* GH18 chitinase

Given the scarce structural information available on chitinases in *Leishmania* and to evaluate their potential as therapeutic targets, the Lbr_ChGH18 (LbrM.16.0800) was modeled in silico using AlphaFold [45] and Phyre2 [46]. AlphaFold models were selected for further analyses owing to their higher predicted accuracy. Models were refined via GalaxyWeb [47] until no further improvements were observed in the Ramachandran-favored parameter [48], reflecting the quality of the protein conformation based on the Ramachandran plot. Model validation was additionally performed using ProSA [49], with Z-scores indicating the overall structural quality relative to experimentally determined protein structures. The three-dimensional structure of Lbr_ChGH18 was visualized and analyzed using UCSF ChimeraX [50, 51].

In this exploratory study, molecular docking was performed with four ligands from the PubChem database [49]. Chitin hexamer (GlcNAc_6_; 5,288,896) was used as a substrate-like ligand, and closantel (42,574), a known natural chitinase inhibitor, served as reference controls. The main focus of the study was on the chitinase-inhibiting peptides argifin (449,124) and argadin (49,123), which were docked to model their interactions within the Lbr_ChGH18 model using CB-Dock2 webserver [52]. Residues involved in the interactions were identified for each ligand, and binding affinities were evaluated using the Vina scoring functions (kcal/mol). Docking poses were ranked based on their Vina scores, which correspond to the estimated binding free energy (ΔG), with lower ΔG values indicating stronger predicted binding affinity.

To better understand the potential inhibitor binding regions, we first performed docking studies using GlcNAc_6_ with Lbr_ChGH18. This preliminary step served as a structural guide to identify substrate-binding motifs (SBMs I-III) and define the size and location of search spaces for subsequent inhibitor docking. We selected the three above-mentioned molecules for our docking analyses based on their pharmacological profiles and potential applications. Closantel was included because of its established use in veterinary medicine against parasitic infections [53] and its well-known role as a chitinase inhibitor in filarial parasites [54], while argifin and argadin were selected because previous studies have demonstrated their low toxicity in human and mouse cellular models [55, 56], and some studies have experimentally demonstrated that they act as competitive inhibitors of chitinases from other organisms [57, 58].

### Prediction of B- and T-cell epitopes in GH18 chitinases of clinically relevant *Leishmania* species

Because of the high conservation and extracellular release of GH18 chitinases in *Leishmania*, their potential antigenicity was evaluated through in silico epitope prediction. The computational tools BepiPred 2.0 and ABCpred [59] were employed to predict potential B-cell linear epitopes. The score threshold for the BepiPred v2.0 was set to 0.55 (the default value is 0.50). The residues with scores above 0.55 were predicted to be part of an epitope. GlycoEP and NetNGlyc were used to detect potential glycosylation sites, based on sequence features and neural network–based predictions, respectively [60]. To minimize prediction bias, only epitopes and glycosylation sites predicted concordantly by at least two independent algorithms were retained for downstream analysis.

For T-cell epitope prediction, the IEDB MHC Binding Prediction Tool [61] was employed to identify peptides capable of binding to major histocompatibility complex (MHC) class I and class II molecules. The Consensus prediction method was selected [61–63], which integrates multiple algorithms (NN-align, SMM, and CombLib) to improve prediction accuracy and reduce methodological bias. To maximize potential population coverage, we used the IEDB-recommended reference panel of 27 alleles for each MHC class. This panel captures the most frequent Human Leukocyte Antigens (HLA) specificities across global populations and is widely adopted in immunoinformatics-based vaccine and epitope discovery studies.

## Results

### Identification and bioinformatic characterization of chitinase genes in kinetoplastids

Bioinformatic analyses of genes encoding chitinases in parasitic and free-living kinetoplastids and various other free-living protist revealed the presence of both mono- and multidomain enzymes, belonging to the glycoside hydrolase families GH18, GH19, and GH20 (Supplementary Tables 1 and 2). In these organisms, chitinase enzymes were predicted to localize in diverse subcellular compartments, suggesting potential functional diversification. Furthermore, several species were found to encode multiple isoenzymes, some of which correspond to putative “dead” chitinases, as concluded from substitutions and deletions of essential catalytic site residues. The chitinase catalytic domain was often associated with one or more additional structural modules, including a membrane-binding domain, a reverse transcriptase (RT_Nltr) domain, a chitin-binding, a non-catalytically active domain (CBD3), a Src homology 3 (SH3b) domain, and GH20b2 β-hexosaminidase domain-like, supporting modular enzyme architectures and potential multifunctionality.

In free-living organisms such as *Naegleria gruberi*, both GH18- and GH19-type chitinases were identified, some containing predicted inactive domains. In *Euglena gracilis*, a single multidomain chitinase was detected, composed of alternating CBD3 and GH18 catalytic domains. In *Diplonema papillatum*, nine mono- and multidomain chitinases were identified, several of which exhibited predicted inactive catalytic sites. In *Bodo saltans*, a free-living kinetoplastid, only GH18 and GH20 chitinases were detected. In *Leishmania* spp., a single GH18-type chitinase gene, highly conserved across species, was identified and located on chromosome 26. Notably, the chitinases of all *Leishmania* species contain no additional domains beyond the catalytic chitinase domain (Supplementary Tables 1 and 2). In contrast, among trypanosomes, only GH20-type chitinases were detected in *Trypanosoma melophagium* and *Trypanosoma theileri* both exhibiting multidomain organization. *T. melophagium* encodes seven GH20 chitinases of similar molecular weight, suggesting gene duplication and functional redundancy. No GH18 or GH19 chitinases were detected in *T. cruzi*, *T. brucei*, or other trypanosomes.

In addition to cytosolic localization, many of these enzymes were predicted to occupy other subcellular compartments (Supplementary Tables 1 and 2). Free-living species exhibited predominantly extracellular chitinases and often also localization in the endoplasmic reticulum (ER) and Golgi apparatus (GA), whereas the enzymes of obligate parasites, in addition to being extracellularly and in the ER–GA, are sometimes also predicted to localize to other subcellular compartments. In *Leishmania* and related subgenera, chitinases are localized primarily to the ER–GA from where they can subsequently be secreted into the extracellular space. *Trypanosoma* species exhibited broader profiles, including the plasma membrane and lysosomes, whereas *Endotrypanum* also showed localization to the mitochondrion and nucleus. In endosymbiont-bearing trypanosomatids, localization was restricted to the ER and extracellular space. Taken together, the data show that in the protists analyzed, several isoforms were predicted to localize simultaneously in multiple compartments, with some distributed across four to five distinct organelles, suggesting a high degree of functional and spatial versatility, potentially supporting diverse intracellular and extracellular roles.

### Multiple alignment, comparison, and phylogenetic analysis of chitinase sequences

**Fig. 1:**
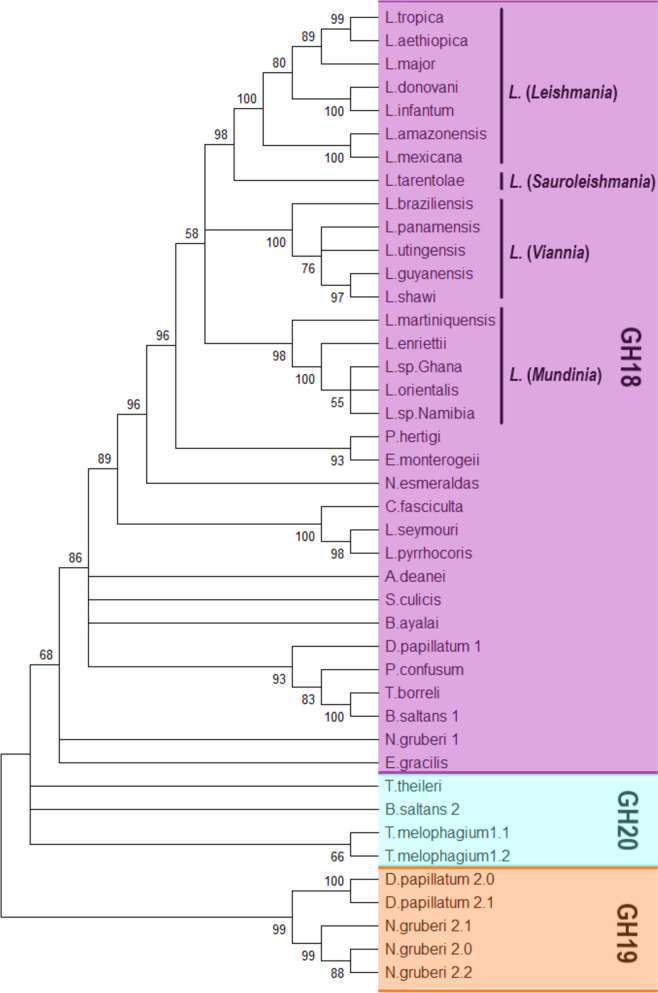
39

An unrooted phylogenetic tree was constructed to study the evolutionary relationships among the chitinase families in the phylum Euglenozoa. The tree (Fig. 1) was constructed using 42 aligned chitinase-domain sequences from different members of the euglenozoan classes Kinetoplastea, Diplonemea, and Euglenida, as well as three sequences of the amoeboflagellate *Naegleria gruberi*.

The analysis shows a high level of conservation of the single-copy gene-encoded GH18 family chitinase in all members of the *Leishmania* genus; their sequences formed a strongly supported monophyletic clade, consistent with previously reported taxonomy classification [64]. The GH18 chitinases from the *Leishmania* species showed segregation into clusters, each one corresponding with its respective subgenus (Leishmania, Sauroleishmania, Vianna, and Mundinia) and those of the Paraleishmania group (*Porcisia hertigi* and *Endotrypanum monterogeii*) [64]. Also, GH18 chitinase proteins are conserved in other members of the Kinetoplastea, including *Trypanoplasma borreli*, a fish-parasitizing bodonid, clustering with the GH18 sequence of the free-living bodonid *B. saltans.* This latter species contains, in addition to its GH18 (called B. saltans 1), two chitinases of the GH20 family. However, only one of these GH20 sequences (called B. saltans 2) was used in the phylogenetic analysis.

Interestingly, multiple chitinase-like genes were detected in *D. papillatum,* including chitinases from the GH18 (four genes) and GH19 (five genes) families (Supplementary Table 1). For phylogenetic inference, one representative sequence from each family was included. Each one formed a group with other enzymes from its respective family. Furthermore, distantly related is the GH18 chitinase of *E. gracilis* (Euglenida), since it shares only low sequence similarity with all its homologs from the Kinetoplastea class. In addition, *N. gruberi* (phylum Heterolobosea) contains a GH18 chitinase and four sequences of the GH19 family; both latter enzymes are grouped in a clade with GH19 chitinases from *D. papillatum*. Interestingly, the GH19 family, which is classically associated with plants, is only sporadically reported in protists.

In contrast with other kinetoplastids, GH18 chitinases are absent from all members of the *Trypanosoma* genus [15]. However, two sequences of chitinases GH20 family were found in *T. theileri* and *T. melophagium* which are closely related to the GH20 from *B. saltans*. Notably, *T. melophagium* harbors seven GH20 gene copies; six of the encoded proteins are 100% identical and share 88.6% identity with the seventh one. Therefore, only two different sequences were used for the phylogenetic analysis. To our knowledge, this study reports for the first time the presence of GH20 chitinases in *T. theileri and T. melophagium*. All these results indicate that, in Euglenozoa, there are patterns of presence, absence, or coexistence of the chitinase families GH18, GH19, and GH20. Interestingly, enzymes of these three chitinase families perform related chitinolytic functions, but do not show an obvious sequence similarity within the euglenozoan phylum.

**Fig. 2:**
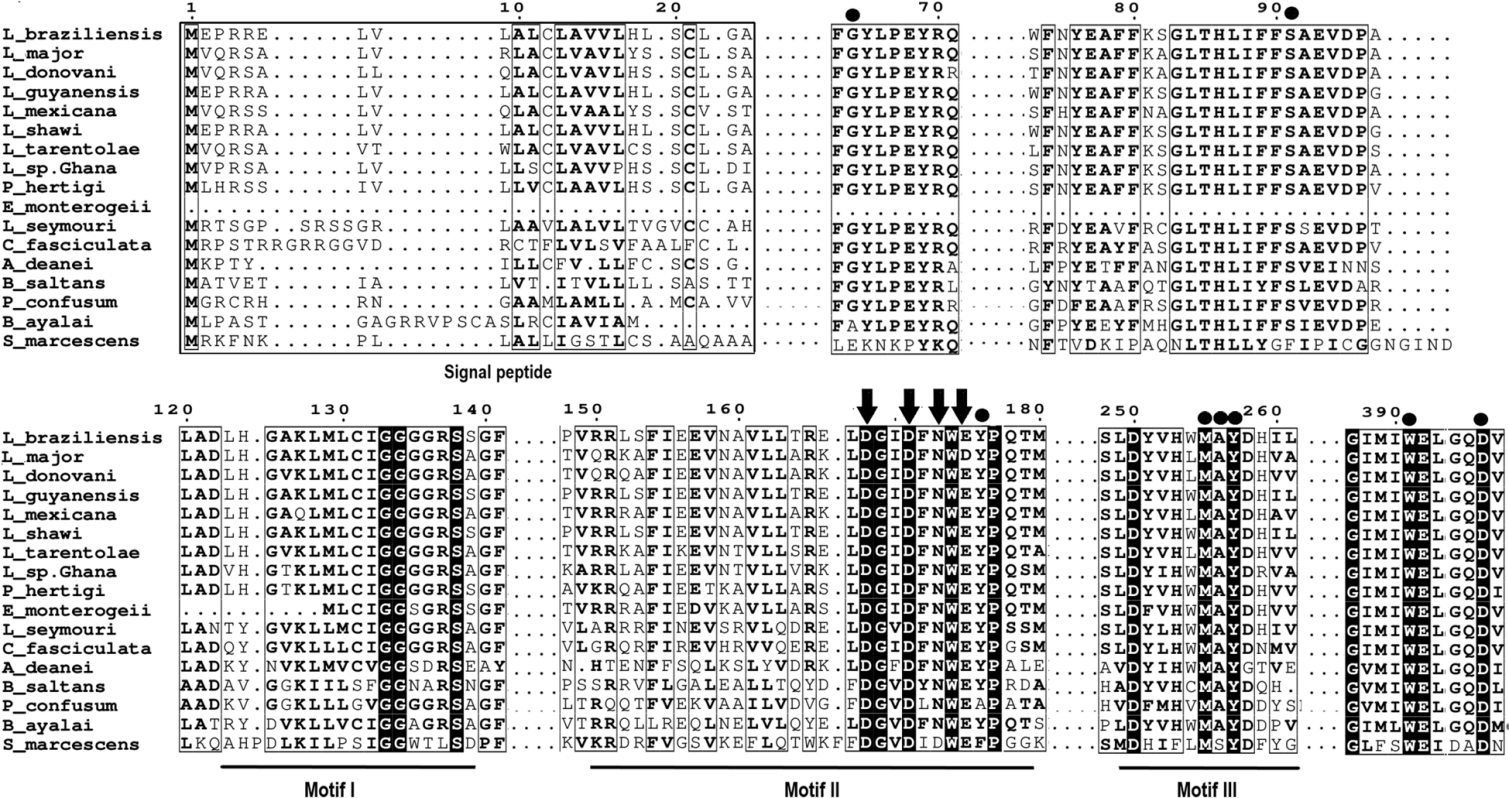
Amino-acid sequence alignment of GH18 chitinase domains. After alignment with the program T Muscle [99], all sequences were analyzed with the online server ESPript 3.0 [100]. The figure shows the sequence of the chitinase of *L. braziliensis* (Lbr_ChGH18) aligned with the corresponding domain of other GH18 chitinases**.** Black boxes indicate residues that, in all sequences, are identical. White boxes indicate residue positions where only conservative substitutions are found, and dots indicate the absence of amino acids at corresponding positions. The highly conserved signal peptide (SP) at the N-terminus is indicated by a rectangular box, followed by the underlined residues of the GH18 family motifs: I (123 – 139), II (150 – 180; catalytic signature DXXDXNWE, 168–176), and III (249 – 261). Residues involved in chitin binding are indicated with black circles. Residues involved in the catalytic/active site are indicated by down-pointing arrows at the top. The following sequences were included in the analysis: *L. braziliensis* (LbrM.16.0800), *L. major* (LmjF.16.0790), *L. donovani* (LdBPK_160790.1), *L. guyanensis* (A0A1E1IUT6_LEIGU), *L. mexicana* (LmxM.16.0790), *L. shawi* (Q4I32_002489), *L. tarentolae Parrot* (LtaP16.0770), *L. sp. Ghana* (GH5_06168), *P. hertigi* (JKF63_05287), *Endotrypanum monterogeii* (EMOLV88_160012400), *L. seymouri* (Lsey_0068_0030), *C. fasciculata* (CFAC1_120016000), *A. deanei* (ADEAN_000766800), *P. confusum* (PCON_0062580), *B. ayalai* (Baya_138_0020), *S. marcescens* (P07254.3)

Consistent with the phylogenetic clustering shown in Fig. 1, the enzyme contains a highly conserved catalytic signature, the **DXXDXDXE** motif (**D**GI**D**FNW**E**), which is characteristic of chitinases and essential for chitin-degrading activity. This conserved motif suggests that the chitinase of *L. braziliensis* (Lbr_ChGH18), encoded by *LbrM.16.0800*, retains the canonical structural and functional features necessary for substrate recognition and catalysis (Fig. 2). Although this motif is highly conserved across all *Leishmania* species, a substitution (D → N of the third aspartate of the signature to asparagine) is observed in this species. The chitinase from *L. mexicana* (Lmex_ChGH18), which also contains this substitution, is known to possess functional chitinase activity [14]. Additionally, the chitinases in *Leishmania* contain a highly conserved signal peptide (SP) and three substrate-binding motifs (SBM I-III): motif I (123–139), motif II (150–179), and motif III (249–262) (Fig. 2).

An important aspect of the chitinase domains of these parasites is that they not only resemble the well-characterized chitinase domains of other organisms, such as that of the bacterium *Serratia marcescens*, but that the sequences within the *Leishmania* chitinase and related kinetoplastid (Chi-Kts) ensemble are also well conserved. Furthermore, a similar level of conservation (76.3–99.1%) is observed among the chitinase domains of these different *Leishmania* chitinase sequences.

Alignments of GH19 chitinase domains from *D. papillatum*, *N. gruberi*, and the extensively characterized GH19 from *Streptomyces alfalfae* revealed a clear conservation of the signature residues characteristic of chitinases of this family (Supplementary Fig. 1A). The putative catalytic sites were strictly conserved across all sequences, while substrate-binding residues exhibited a high degree of conservation, suggesting functional maintenance of enzymatic activity. Particularly, the *N. gruberi* sequence displayed minor variations in non-catalytic regions, reflecting potential lineage-specific diversification. Similarly, alignment of GH20 chitinases from *B. saltans*, *T. theilieri*, *T. melophagium*, and the well-characterized GH20 from *S. marcescens* also highlighted the strict conservation of residues directly involved in catalysis and substrate binding (Supplementary Fig. 1B). Conservation patterns observed in the GH19 and GH20 alignments, particularly in the active site and substrate-binding regions, underscore the evolutionary pressure to maintain enzymatic function, despite taxonomic divergence among the analyzed species.

### RNA-seq data compilation and expression profiling of GH18 chitinase in *L. braziliensis*

**Fig. 3:**
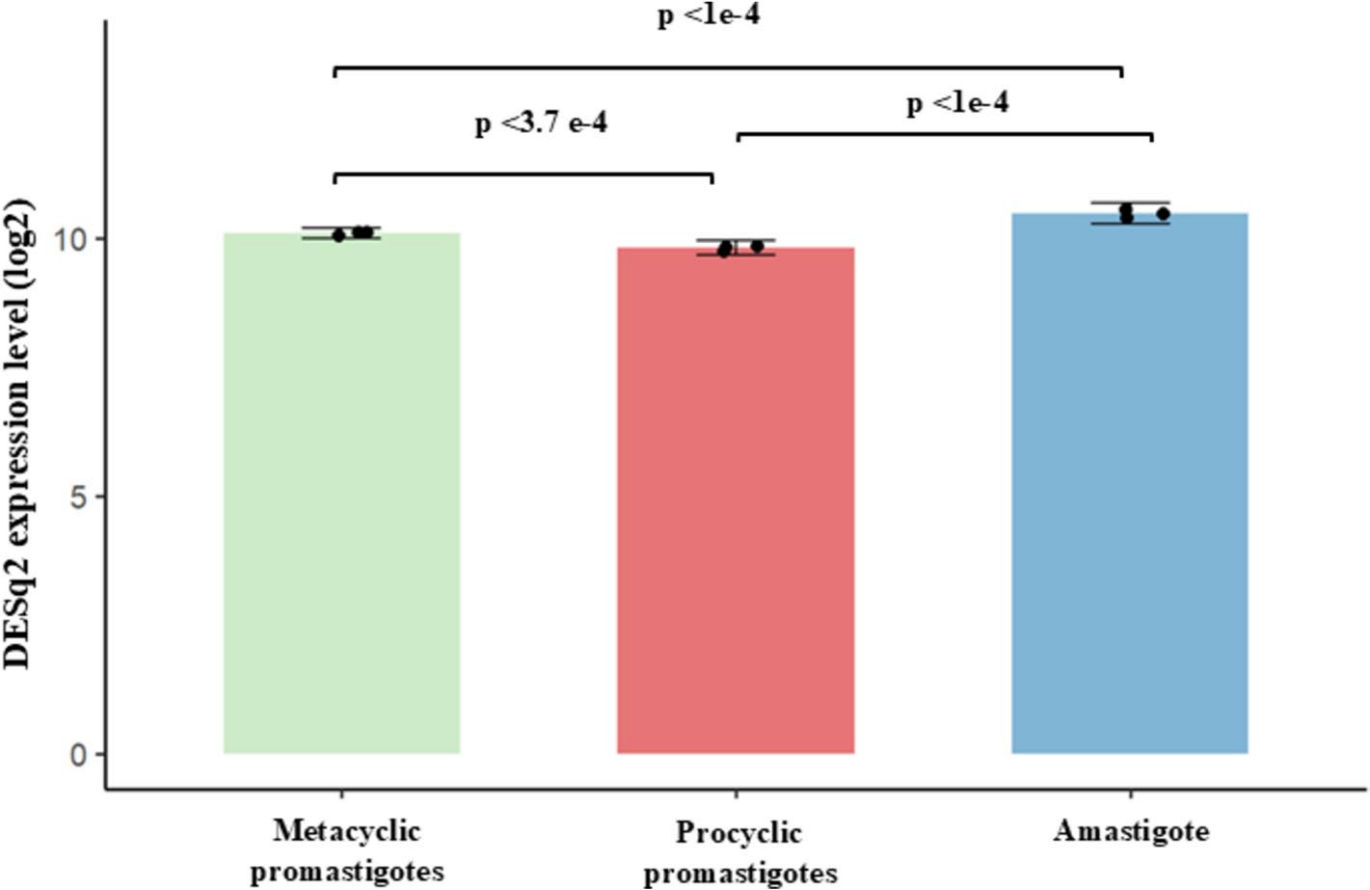
Expression analysis of the *LbrM.16.0800* gene across developmental stages. Differential expression analysis performed using DESeq2 on RNA-seq data from three biological replicates per stage. Raw read counts were normalized using the median-of-ratios method (size factor normalization) implemented in DESeq2 and are shown as log₂-transformed normalized counts. Statistical significance was determined using an adjusted *p*-value < 0.05

The chitinase gene *LbrM.16.0800* showed a modest, albeit statistically significant increase in expression in amastigotes compared with both procyclic and metacyclic promastigotes, as determined by DESeq2 differential expression analysis (Fig. 3).

### Structure modeling and docking analyses of the *L. braziliensis* GH18 chitinase

The three-dimensional structure of the Lbr_ChGH18 was predicted de novo from its amino-acid sequence using AlphaFold (Supplementary Fig. 2). The model was subsequently refined using GalaxyWeb and exhibited a Ramachandran-favored value of 98.90% (Supplementary Table 3) and a Z-score of −7.57 in ProSA, reflecting high stereochemical quality (Supplementary Fig. 3A). This analysis revealed that Lbr_ChGH18 adopts the canonical chitinase fold, containing a TIM-barrel (β/α)_8_ domain typical of GH18 family enzymes. Structural inspection revealed that the catalytic residues are located at the bottom of a deep substrate-binding groove, forming the characteristic cleft for chitin interaction (Fig. 2A). The overall fold and catalytic motif of Lbr_ChGH18 are highly conserved, displaying strong structural similarity to previously experimentally characterized GH18 chitinases, including chitinase A from *Serratia marcescens* (*Sm*ChiA) (Supplementary Fig. 4A). When the structure of Lbr_ChGH18 was superimposed onto *Sm*ChiA, the domains aligned almost perfectly, highlighting a remarkable conservation of the overall fold and architecture of the catalytic site (Supplementary Fig. 4B).

**Fig. 4:**
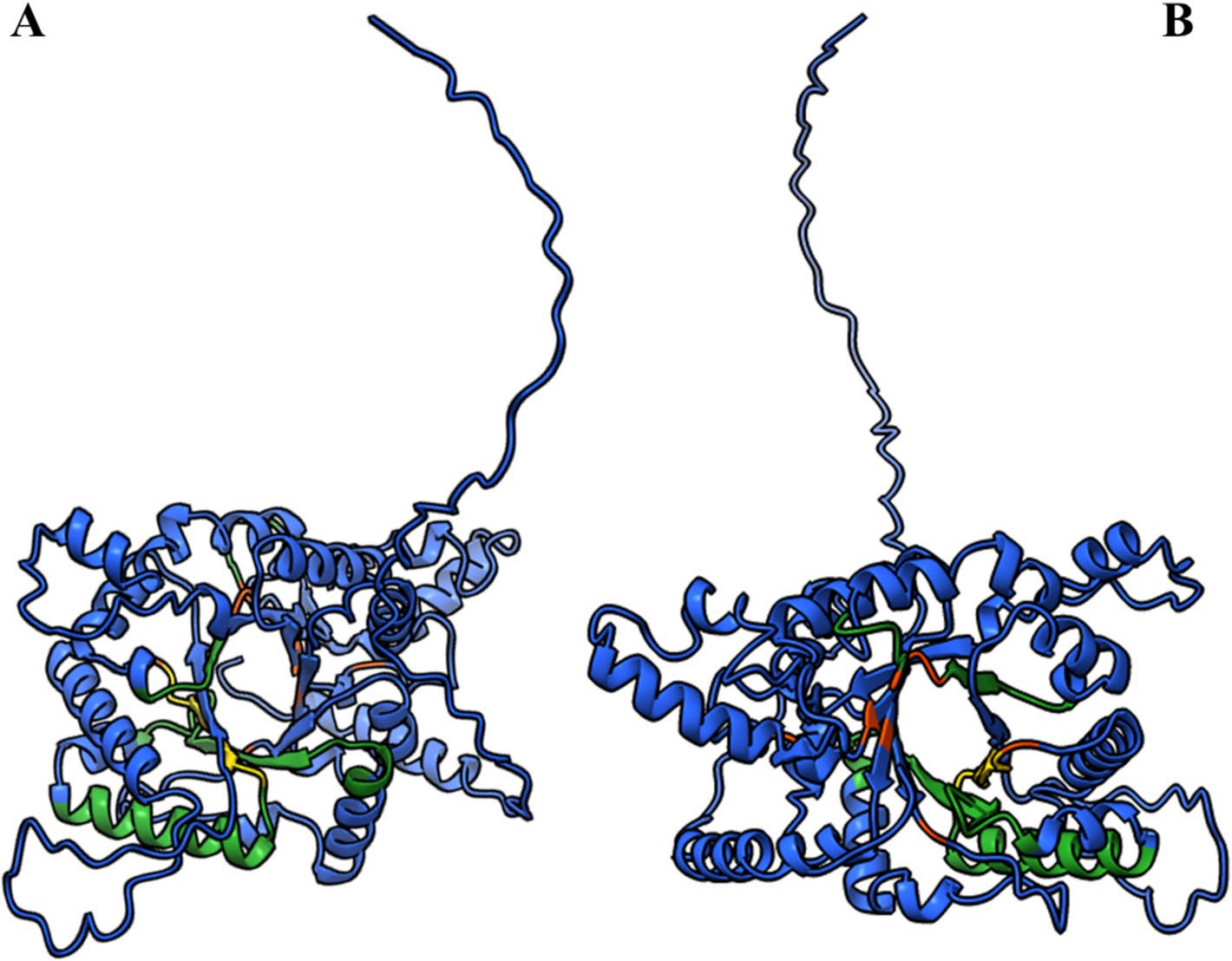
Structure modeling of the processed chitinase from *L. braziliensis* (pLbr_ChGH18). Front **A** and rear **B** views of the processed enzyme (pLbr_ChGH18) after signal peptide removal, representing the processed form used for subsequent docking analyses. Highlighted parts correspond to the residues of the conserved DXDXXDXE catalytic motif (yellow), residues forming the chitinase domain (red), and substrate-binding motifs (SBM I-III) (green)

We also modeled the Lmx_ChGH18, a structure not previously reported, which also exhibited the typical GH18 fold. Superimposition of Lmx_ChGH18 onto Lbr_ChGH18 (75.8% percent identity) revealed an almost perfect overlap of the domains, demonstrating that these two New World *Leishmania* species preserve the canonical GH18 architecture of the catalytic site (Supplementary Fig. 5A and 5B). Although the initial structure model included the N-terminal signal peptide (SP) (Supplementary Fig. 2), experimental evidence from *L. mexicana* and *L. donovani* indicates that their chitinases are secreted [16, 19]. Therefore, to represent the biologically relevant form, the predicted SP was removed, and the processed chitinase structure was modeled, hereafter referred to as pLbr_ChGH18 (Fig. 4). The refined pLbr_ChGH18 exhibited 98.80% of its residues in Ramachandran-favored regions (Supplementary Tables 5 and 6) and a ProSA Z-score of − 8.46 (Supplementary Fig. 3B), indicating a stereochemically structured protein comparable to experimentally determined proteins of similar size.

**Fig. 5:**
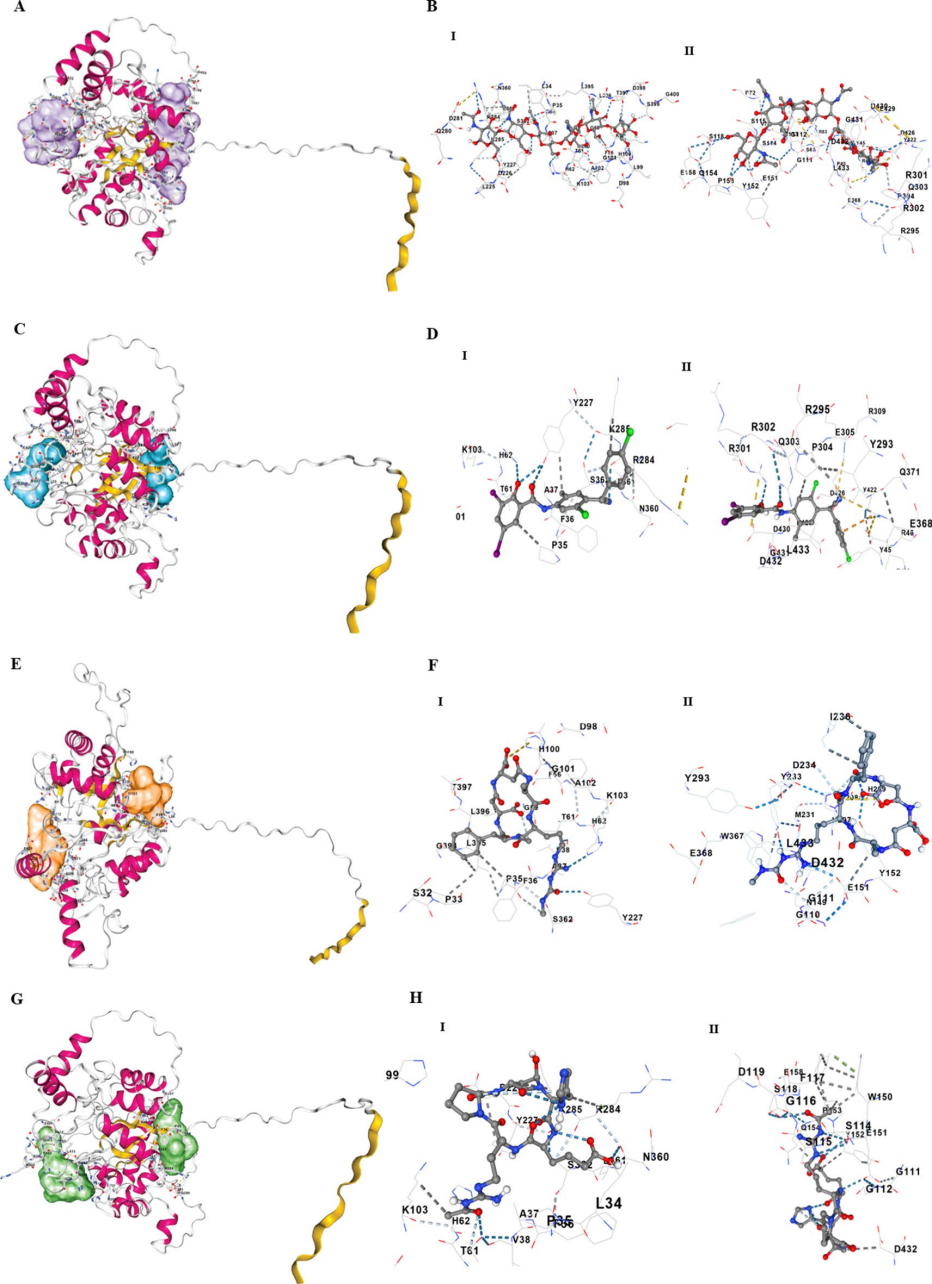
Molecular docking of pLbr_GH18 chitinase with chitin hexamer (GlcNAc_6_) and inhibitors. Panels **A**–**H** show the energetically most favorable binding models of the chitin hexamer (GlcNAc_6_) **A**, **B**, closantel **C**, **D**, argifin **E**, **F**, and argadin **G**, **H** at the two binding sites of the enzyme. Predicted binding affinities (Vina scores) are: GlcNAc_6_ –6.8 ± 0.5 kcal/mol (site I) and −7.2 ± 0.4 kcal/mol (site II); closantel, −7.5 ± 0.3 (site I) and −8.0 ± 0.2 kcal/mol (site II); argifin, −7.4 ± 0.3 (site I) and −7.4 ± 0.2 kcal/mol (site II); and argadin, −7.9 ± 0.3 (site I) and −7.6 ± 0.5 kcal/mol (site II). Interactions include hydrogen bonds (dark blue), weak hydrogen bonds (light blue), hydrophobic interactions (gray), ionic interactions (yellow), cation–π interactions (orange), and π–π stacking (green)

The pLbr_ChGH18 model was subsequently used for molecular docking analyses and to identify potential inhibitor-binding pockets (Fig. 5 panels A, C, E, and G).

The docking results revealed that the modeled pLbr_ChGH18 possesses five distinct binding sites for the chitin hexamer and other ligands (closantel, argadin, and argifin), which are known inhibitors of chitinasases. Each site is accessible through multiple docking poses and exhibits different predicted binding affinities for the chitin hexamer (GlcNAc_6_). These analyses provide critical insights into the mechanism by which the chitinase pLbr_ChGH18 interacts with GlcNAc_6_, an analog of its natural substrate, as well as with the three above-mentioned, well-characterized inhibitors. Two of the five binding sites, located near the N-terminal (site I) and C-terminal (site II) ends of the protein (Supplementary Fig. 6 A–C), may have great physiological relevance, since their locations coincide with the opposite ends of the catalytic barrel, suggesting that the enzyme contains a cleft—a common trait among some GH18 chitinases. The binding of inhibitors at these sites supports the hypothesis that they function as “plugs” at the entrances of the catalytic groove channel, potentially blocking substrate access and product release (Fig. 5, panels A, C, E, and G and Supplementary Fig. 7). The positioning of these inhibitors on the chitinase of Lbr_ChGH18 resembles that observed for chitinases from other species, such as the parasitic nematode *Onchocerca volvulus* and chitinase B of *S. marcescens*, near the catalytic domain at the entrance of the TIM barrel [65, 66]. The surface representation of Lbr_ChGH18 (Supplementary Fig. 6D) revealed that its topology is highly similar to that described for chitinases from other organisms.

The calculated Vina docking scores ranged from −6.4 to −8.0 kcal/mol, indicating moderate to strong binding affinities (Table 1). The GlcNAc_6_ displayed slightly different affinities at the two sites. At site I, it showed an affinity of −6.95 ± 0.38 kcal/mol, whereas at site II the calculated affinity was –8.0 ± 0.31 kcal/mol. Site I consists of at least 41 residues, 21 of them interacting directly with the GlcNAc_6_. Among these, residues D226 and Y227 are part of the substrate-binding motif III (SBM-III) identified in the protein (Fig. 2, Fig. 5, panels A and B, and Table 1). Site II comprises 48 residues, 20 of which interact directly with the hexamer. Residues G111, G112, R113, S114, S115, G116, F117, and S118 are also part of substrate-binding motif I (SBM-I). Additionally, GlcNAc_6_ interacts directly with residues E151, Y152, P153, Q154, and E158, which are part of substrate-binding motif II (SBM-II). SBM-I and SBM-II binding motifs are involved in substrate stabilization during catalysis. Residues located towards the C-terminal end of the protein, including P304, Y422, D426, D432, L433, and G431, also contribute to interactions at site II (Fig. 2 and Table 1).

**Table 1:** Docking analysis of pLbr_GH18 with chitin hexamer (GlcNAc_6_) and inhibitory ligands: Binding energies and residues involved in the ligand-binding sites*

Ligand	Binding site	Vina score (kcal/mol) (Prom. ± SD)	Residues involved in the ligand-binding sites*
Chitin hexamer	**1**	−6.95 ± 0.38	H100 **G101** A102 **K103** L225 **D226 Y227** S32 P33 **L34 P35 F36 A37 V38** F56 K57 S58 G59 L60 **T61 H62 L99** R170 **H199** E279 Q280 **D28**1 H282 **R284 K285** L286 **N360 L361** S362 H387 L390 **L395 L396 T397** D398 S399
	**2**	−8.00 ± 0.31	**G111 G112 R113** S114 S115 G116 F117 S118 **E151 Y152** **Y233 D234 H235 I236** P43 **E44 Y45** R46 F66 S67 **E69 P72** H79 **R83** N149 P153 Q154 E158 F162 R295 R300 R301 R302 Q303 **P304** E305 T306 E368 Q371 Y422 **D426** V427 A428 **E429 D430 G431 D432 L433**
Closantel	1	−7.5 ± 0.3	H100 **G101 K103** **H199 D226 Y227** **L34 P35 F36 A37 V38 T61 H62 R284 K285 N360 L361 S362 G363 L395 L396**
	2	−8.0 ± 0.2	**G110 G111 G112 R113** N149 **E151 Y152** **M231 Y233 D234 I236** **Y45 R46 E69 R83** H207 Y293 **R295 R301** Q303 P304 E305 T306 R309 E368 Q371 **D426** V427 **E429 D430 G431 D432 L433**
Argifin	1	−7.4 ± 0.3	L225 **D226 Y227** **G101 K103** S32 P33 **L34 P35 F36 A37 V38 T61 H62 H199** V201 A286 **N360 L361** S362 **G363 L395 L396** T397
	2	−7.4 ± 0.2	**G111 G112 R113** S114 S115 N149** E151 Y152** H207 **M231 Y233 D234 H235** E44 **Y45 R46** F66 S67 **E69** V70 P72 H79 **R83 R295** R300 R301 R302 **P304** E305 T306 R309 W367 E368 Q371 Y422 **D426 E429 D430 G431 D432 L433**
Argadin	1	−7.9 ± 0.3	H100 **G101** V102 **K103** L225 **D226 Y227** S32 P33 **L34 P35 F36 A37 V38** F56 K57 G59 L60 **T61 H62** L99 **H199** V201 D281 **R284 K285 N360 L361 S362 G363** L390 G394 **L395 L396 T397**
	2	−7.6 ± 0.5	G110 **G111 G112** R113 S114 S115 **N149 W150 E151 Y152** M231 Y233 D234 H235 I236 P43 **E44 Y45** F66 S67 **E69** H79 **R83** P153 Q154 A205 L206 H207 P208 H209 Y247 **R295 R301** R302 W367 **E429 D430 G431 D432 L433**

^*^The amino-acid numbering was assigned according to their position after protein processing (pLbr_ChGH18)

Residues in bold: Common residues involved in the interaction of chitinase with the four ligands

Residues underlined with a dotted line (R): Part of substrate-binding site I

Residues underlined with a solid line (R): Part of substrate-binding site II or catalytic site DXXDXDXE

Residues underlined with a dashed line (R): Part of substrate-binding site III

Additionally, GlcNAc_6_ interacts directly with residues E151, Y152, P153, Q154, and E158. Notably, E151 is part of the catalytic site, whereas Y152 is one of the residues involved in chitin binding. E151 functions as the catalytic acid–base residue that promotes catalysis through a mechanism known as substrate-assisted catalysis, reported for *Sm*ChiA and other GH18s. In this process, the enzyme moves back and forth along the chitin chain, functioning like a molecular motor driven by the hydrolysis of the substrate [5, 67] (Supplementary Fig. 8). In pLbr_ChGH18, the highly conserved glutamic acid residue (E151), located on the TIM barrel, protonates a glycosidic oxygen of the chitin hexamer (an acid/base reaction), initiating the cleavage of the glycosidic bond. Subsequently, the carbonyl group of the neighboring N-acetylglucosamine unit cleaves the bond through the formation of an oxazolinium ion, which is stabilized by residue N149 via hydrogen bonding and polarization interactions (fulfilling the role of a D residue) (Supplementary Fig. 8). Then, E151 in its anionic form, abstracts a proton from a water molecule adjacent to the oxazolinium ion, which is then attacked by the hydroxide ion, thereby completing the hydrolysis.

The difference in affinity between sites I and II could reflect the directionality of processing: site I could represent the initial binding position, with lower affinity, while site II offers greater stabilization, favoring substrate translocation along the catalytic groove during catalysis.

Furthermore, closantel exhibited slightly different affinities for the two sites: site I (−7.5 ± 0.3 kcal/mol) compared to site II (−8.0 ± 0.2 kcal/mol), with the strongest interaction observed at site II. Site I is composed of at least 21 residues, 20 of which interact directly with closantel. Some residues, such as H100, G101, and K103, are part of SBM-I, while D226 and Y227 belong to SBM-III (Fig. 2, Fig. 5D, and Table 1). At site II, at least 33 residues are involved, 19 of which interact directly with this molecule. Similar to GlcNAc_6_, this ligand also interacts with residues G110, G111, G112, and R113 (SBM-I). Residues located towards the C-terminal end of the protein and outside of the substrate-binding motifs, including R301–E305, D426–V427, and E429–L433, also contribute to interactions at site II (Fig. 2, Fig. 5D, and Table 1). Other residues, such as N149, E151, and Y152, may be part of the substrate-binding motif II (SBM-II), and M231, Y233, D234, H235, and I236, relevant for binding within SBM-III, but do not interact directly with closantel.

Notably, argadin and argifin showed comparable affinities across both sites (Table 1). For argifin, the Vina scores were −7.9 ± 0.3 kcal/mol at site I and −7.6 ± 0.5 kcal/mol at site II. Site I is composed of at least 24 residues, 17 of them interacting directly with argifin. Residues L225, D226, and Y227 are part of SBM-III, while G101 and K103 are part of SBM I. At site II, at least 41 residues are involved, 18 of which interact directly with argifin. Residues G111, G112, R113, S114, and S115 belong to SBM-I, while others, such as N149, E151, and Y152, are part of the DXXDXNXE motif within SBM-II (Fig. 2, Fig. 5F, and Table 1).

For argadin, the Vina scores were −7.4 ± 0.2 kcal/mol at site I and −7.4 ± 0.3 kcal/mol at site II (Table 1). Site I contains at least 35 residues, 20 of which interact directly with argadin. Residues such as H100, G101, V102, and K103 belong to SBM-I, while D226 and Y227 are part of SBM-III (Fig. 2, Fig. 5D, and Table 1). At site II, at least 40 residues are involved, 17 of them interacting directly with this ligand, including residues G110, G111, G112, R113, S114, and S115 (SBM-I). Residues N149, W150, E151, and Y152, situated within SBM-II, also contribute to interactions (Fig. 2, Fig. 5H, and Table 1). Residues M231, Y233, D234, H235, and I236 (SBM-III) also form part of site II. The small differences in these values indicate that each of these three synthetic inhibitors has a comparable binding affinity for sites I and II.

These analyses show that the three inhibitors studied interact with conserved residues present in the substrate-binding motifs of pLbr_ChGH18. Key residues in SBM-I (H100, G101, K103, G110–R113, S114–S115), SBM-II (N149, W150, E151, and Y152), and SBM-III (L225, D226, Y227, M231, Y233, D234, H235, I236) are consistently involved in binding, highlighting conserved interaction interfaces. Residues at the C-terminal end of the protein and outside of the substrate-binding motifs (including R301–E305, D426–V427, and E429–L433) play a significant role in closantel binding. At binding site I, 17 residues are consistently involved in interactions with all four ligands, whereas at site II, 18 residues are commonly engaged across the ligands (Table 1; Fig. 5 panels C, E, and G).

Furthermore, the dual binding pattern of argifin and argadin indicates that these ligands interact not only with key residues of the catalytic and substrate-binding sites occupied by GlcNAc_6_ and closantel but also with adjacent regions. These interactions occur through hydrogen bonds, ionic, hydrophobic, and stacking contacts, thereby contributing to the overall stability of ligand binding within these sites (Table 1 and Fig. 5 panels B, D, and F). Interestingly, at site II, which has the largest volume, closantel showed the most negative Vina score (−8.0 ± 0.2 kcal/mol), suggesting the strongest site affinity. Nonetheless, it has fewer contact residues, indicating that the interactions must be stronger.

At this site, the interactions observed for closantel were primarily: (a) hydrogen bonds through residues R295 and R301; and b) stacking interactions between the phenolic and pyrrolidine rings of residues Y293 and P304, respectively, with the delocalized π cloud of the aromatic rings and the –C≡N group of the ligand (Fig. 5D). Thus, these results highlight that both types of moieties have the potential to be considered in the design of new molecules with improved affinity for this chitinase.

These docking studies were also performed using the precursor protein structure, Lbr_ChGH18, and the resulting Vina scores were very similar to those obtained with pLbr_ChGH18 (results not shown). This indicates that the removal of the signal peptide does not substantially affect protein–ligand interactions.

These results suggest that argifin and argadin can occupy residues within the substrate-binding sites and those associated with the catalytic site of the enzyme, most likely acting as competitive inhibitors of the *Leishmania* enzyme, in agreement with results from experimental studies of chitinases from other organisms [57, 58].

### Prediction of B-cell epitopes in GH18 chitinases of clinically relevant *Leishmania* species

Comparative analysis revealed B-epitopes conserved across all New World *Leishmania* species, indicating regions of structural and antigenic similarity within this phylogenetic group. GGGGRSSGFSDL, NLSGIMIWE, PMSLMAAVHEQLAD, and TDSGHGSGGDVNGD (or variants) are epitopes highly conserved across all New World *Leishmania* (Table 2 and Supplementary Table 6). Epitope NLSGIMIWE, through its motif NLSG, overlaps with a predicted glycosylation site (N*LSG), suggesting that post-translational modifications may influence their accessibility and immunogenicity. Subgenus-specific epitopes were identified. Epitopes such as VAAAASAAVRDSAAS…/…SSSQNASIT were highly conserved within the Viannia subgenus and correspond to a predicted glycosylated region (N*ASIT). In contrast, TAIGSGHNTSIT, KNHPTWV, and NTSIAE were exclusively detected in the species of the Leishmania subgenus. The N*TSI motif represents a predicted N-glycosylation site, suggesting potential modulation of epitope exposure and immune recognition. All predicted epitopes exhibited BepiPred 2.0 scores ≥ 0.55 and ABCpred scores ranging from 0.68 to 0.95, indicating a high probability of antigenicity (Supplementary Table 6). These findings provide a basis for identifying antigenic regions with potential interspecies relevance in the New World, as well as subgenus-specific epitopes. Importantly, epitopes NLSGIMIWE, MSLMTAVHEQLA, and SPDVAE are highly conserved and shared between *Leishmania* species from both the Old and New Worlds, featuring an identical predicted glycosylation motif (N*LSG) (Supplementary Tables 6 and 7). This latter motif has also previously been reported in *L. mexicana* [14].

**Table 2:** Predicted linear B-cell epitopes and potential glycosylation sites in new World *Leishmania* chitinases

Peptide #	Epitope	Present in species	Glycosylation site (if any)	Associated subgenus	Conserved in old World *Leishmania*
1	GGGGRSSGFSDL	*L. braziliensis*, *L. panamensis*, *L. guyanensis*, *L. mexicana*, *L. amazonensis*	–	Highly conserved across all New World Leishmania	No
2	NLSGIMIWEL	*L. braziliensis*, *L. panamensis*, *L. guyanensis*, *L. mexicana*, *L. amazonensis*	N*LSG	Highly conserved across all New World Leishmania/ Predicted glycosylation	Yes
3	PMSLMAAVHEQLAD	*L. braziliensis*, *L. panamensis*, *L. guyanensis*, *L. mexicana*, *L. amazonensis*	–	Highly conserved across all New World Leishmania	Yes
4	TDSGHGSGGDVNGD (or variants)	*L. braziliensis*, *L. panamensis*, *L. guyanensis*, *L.mexicana*, *L. amazonensis*	–	Highly conserved across all New World Leishmania	No
5	SPDVAE	*L. braziliensis, L. panamensis, L. guyanensis, L.mexicana, L. amazonensis*	–	Highly conserved across all New World Leishmania	Yes
6	VAAAASAAVRDSAAS… / …SSSQNASIT	*L. braziliensis*, *L. panamensis*, *L. guyanensis*	N*ASI	Highly conserved within Viannia/ Predicted glycosylation/ Absent in Leishmania (Leishmania) species	No
7	TAIGSGHNTSIT / NTSIAE	*L. mexicana*, *L. amazonensis*	N*TSI	Present only in the Leishmania (Leishmania)/ Predicted glycosylation/ Absent in Viannia	No
8	KNHPTWV	*L. mexicana*, *L. amazonensis*	–	Specific to Leishmania (Leishmania) Absent in Viannia	No

Epitope prediction was performed using BepiPred 2.0 (threshold 0.55) and ABCpred (cutoff > 0.50). Glycosylation sites were predicted with GlycoEP and NetNGlyc (threshold 0.50), with N* indicating predicted glycosylated asparagine residues

^#^: Peptide number

It is important to note that, unlike New World *Leishmania*, epitopes and glycosylation motifs are highly conserved in Old World species. While New World species display subgenus-specific epitopes, Old World species retain a shared repertoire with some variations (Supplementary Table 7). We also analyzed human chitinases using the same epitope prediction pipelines applied to *Leishmania* chitinases. No similar epitopes were identified, indicating an unlikelihood of cross-reactivity and suggesting that the predicted *Leishmania* epitopes are parasite-specific. This is consistent with the alignment results, which show low sequence identities (15–16%) between human and *Leishmania* chitinases (Supplementary Table 8).

### Prediction of T-cell epitopes in GH18 chitinases of clinically relevant *Leishmania* species

We identified 10 high-confidence MHC-I epitope nonamers (9-mers) with broad HLA coverage. Predicted anchor residues were consistent with canonical HLA-I binding motifs, with hydrophobic amino acids predominantly occupying P2 and PΩ (residue 9), as well as polar or charged residues (Table 3).

**Table 3:**
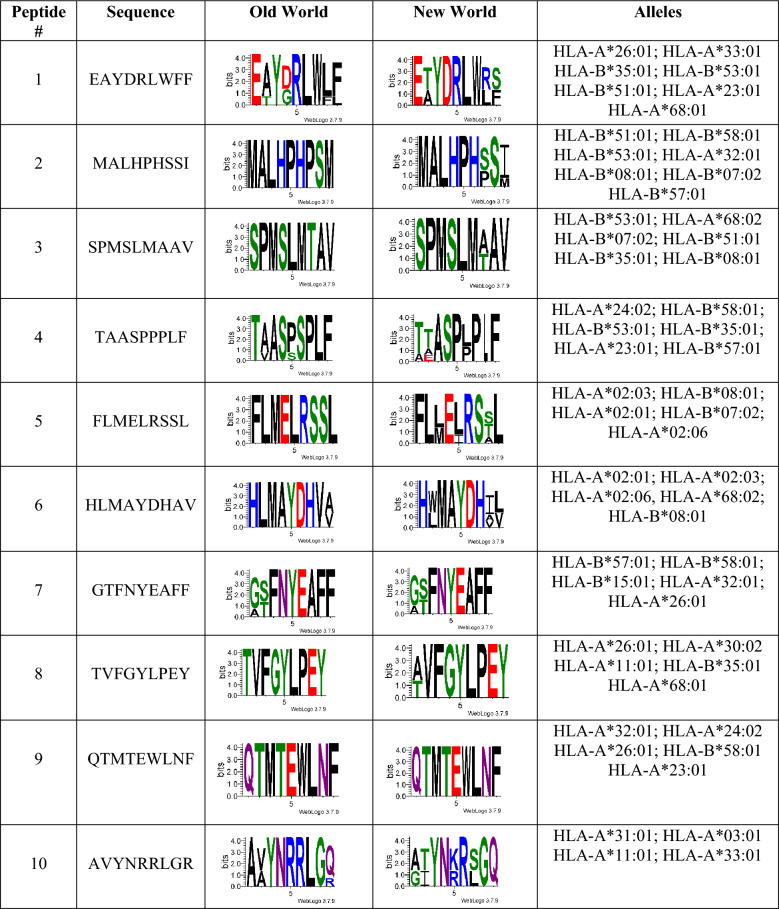
Top 10 predicted MHC class I-restricted epitopes from Old World and New World *Leishmania* chitinases

Sequence conservation is represented using WebLogo (https://weblogo.threeplusone.com/create.cgi [68] and displayed separately for Old World and New World *Leishmania* species

^#^: Peptide number

Each peptide is predicted to bind between four and eight HLA class I alleles, including globally frequent alleles such as HLA-A02 variants, HLA-A23/24/26, and HLA-B35, B51, B53, B57, and B*58. Several peptides (such as EAYDRLWFF (1), MALHPHSSI (2), SPMSLMAAV (3), and TAASPPPLF (4)) exhibited marked promiscuity, simultaneously binding multiple HLA-A and HLA-B molecules. It is important to note that among the 10 selected peptides, only one is conserved (QTMTEWLNF (9)) between Old and New World *Leishmania*. However, others exhibit only minimal variation (SPMSLMAAV (3) and TVFGYLPEY (8)).

We identified 10 high-confidence MHC-II epitopes, all being 15-mer peptides containing predicted binding cores compatible with multiple HLA class II heterodimers (HLA-II) (Table 4). Each peptide is bound between two and four HLA-DQ, HLA-DP, or HLA-DR molecules, including globally frequent alleles such as DPB101:01, DPB102:01, DPB104:01, DQB102/03/06, and DRB18/09.

**Table 4:**
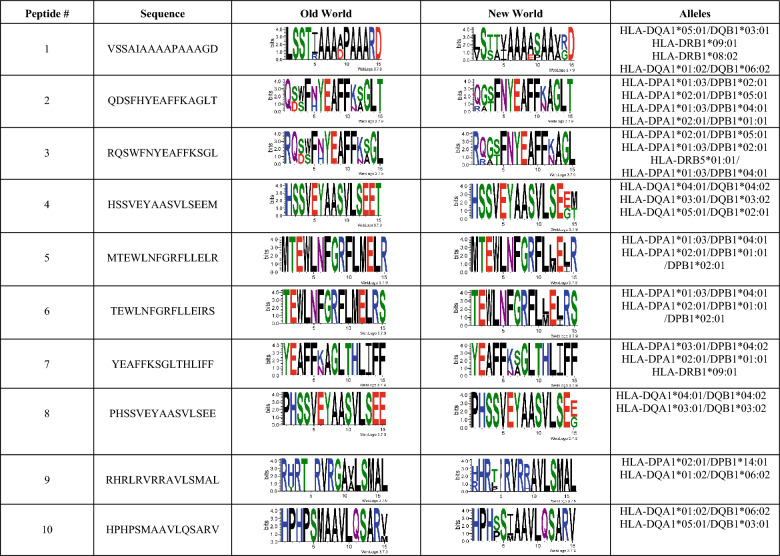
Top 10 predicted MHC class-II-restricted epitopes from Old World and New World Leishmania chitinases

Sequence conservation is represented using WebLogo (https://weblogo.threeplusone.com/create.cgi [68] and displayed separately for Old World and New World *Leishmania* species

^#^: Peptide number

Unlike those observed for MHC-I epitopes, no peptides were strictly conserved in both groups of *Leishmania*. However, some of these, such as MTEWLNFGRFLLELR (#5), TEWLNFGRFLLEIRS (#6) (differing only by a single flanking residue at the N- and C-termini), YEAFFKSGLTHLIFF (#7), and PHSSVEYAASVLSEE (#8), exhibit scarce variation and are predicted to bind to the same HLA-II molecules (DPA1*01:03, DPB1*04:01, HLA-DPA1*02:01, DPB1*01:01, DPB1*02:01, and DRB1*09:01). Several peptides (such as MTEWLNFGRFLLELR (#5) and TEWLNFGRFLLEIRS (#6); HSSVEYAASVLSEEM (#4) and PHSSVEYAASVLSEE (#8); QDSFHYEAFFKAGL (#2), RQSWFNYEAFFKSGL (#3), and YEAFFKSGLTHLIFF (#3)) originated from overlapping regions, revealing three immunodominant hotspots enriched for the shared motifs TEWLNFGRFLLER, HSSVEYAASVLSEE, and YEAFFK, which were recognized by multiple HLA-DP and HLA-DQ molecules. Notably, these epitopes bind multiple major MHC-II loci —DP, DQ, and DR— which are associated with early, chronic/persistent immune responses, and population-dominant polymorphic alleles, respectively.

When comparing Tables 3 and 4 to identify complete overlaps between MHC-I and MHC-II epitopes, no exact matches were observed. However, several partial overlaps were noted: QTMTEWLNF (MHC-I #9) shares the motif “TEWLNF” with MTEWLNFGRFLLELR and TEWLNFGRFLLEIRS (MHC-II #5 and #6). Similarly, GTFNYEAFF (MHC-I #7) contains the sequence “YEAFF,” which is present in QDSFHYEAFFKAGLT, RQSWFNYEAFFKSGL, and YEAFFKSGLTHLIFF (MHC-II #2, #3, and #7).

Importantly, further comparison revealed that some sequences in Table 2, corresponding to B-cell epitopes, partially overlap with identified MHC-I and MHC-II peptides. Specifically, PMSLMAAVHEQLAD B-cell peptide #3 shares the core motif PMSLMAAV with MHC-I peptide #3 (SPMSLMAAV), whereas B-cell peptide #6 VAAAASAAVRDSAAS exhibits partial similarity with MHC-II peptide #1 (VSSAIAAAAPAAAGD).

## Discussion

The principal novelty of this study lies in the comprehensive characterization of GH18 chitinases from *L. braziliensis*. In addition, we report for the first time the presence of GH18, GH19, and GH20 chitinases in other kinetoplastid organisms, providing clues about the inheritance, acquisition and/or losses of these enzymes in kinetoplastid lineages, and insights into their evolutionary relationships, the conservation of functional domains, and their structural and functional diversity, while establishing a framework for future studies on chitinases in these parasites.

Analysis of the genomes of different protists revealed the presence of open-reading frames (ORFs) for chitinase in several species. Some of these ORFs code for unusually large chitinases, including those from *N. gruberi* (D2UZU7_NAEGR), *Euglena gracilis* (A0A2Z5U2B9_EUGGR), *D. papillatum* (KAJ9467433.1 and KAJ9454922.1), and *B. saltans* (BSAL_68100), which appear to contain additional domains fused to the chitinase domain.

In *Leishmania* and other obligate parasites, GH18 proteins are single-domain enzymes, whereas in some free-living protists, they may contain additional associated domains, involved in chitin binding, substrate degradation, microbial adhesion, recognition of bacterial cell wall motifs, or telomere maintenance [69, 70]. Our analysis predicts that some of these chitinases are catalytically inactive or "dead" enzymes, similar to what we have observed for other enzymes in these parasites, as previously reported [71]. These findings suggest functional diversification and possible specialization among kinetoplastid chitinases.

The predicted subcellular localizations of GH18 chitinases in *Leishmania* and other kinetoplastids supports the hypothesis that chitinases in parasitic kinetoplastids can perform both intracellular and extracellular functions related to pathogen-host interactions, survival, immune evasion, infection persistence, or virulence, as has been described in other pathogens [8, 72–74].

Chitinase gene repertoires in kinetoplastids and related protists are highly variable, ranging from a single GH18 in *Leishmania* to multiple GH20 copies in *T. melophagium*. Notably, GH18 was identified in *T. borreli*, where it had not been previously reported [12, 15]. The physiological significance of GH20 duplication in *T. melophagium* remains unknown. However, in trypanosomatids, gene duplication is a central source of genetic diversity, influencing parasite fitness and responses to environmental conditions. Many amplified gene families relate to parasite–host interactions [71]. The detection of GH19 in *Diplonema* and *Naegleria*, a chitinase mostly found in plants [75], highlights the sporadic occurrence of this family in protists, consistent with potential lateral gene transfers [76].

GH18 DXXDXDXE (DGIDFNWE) catalytic motifs are conserved across *Leishmania* and related kinetoplastids. Notably, a D → N substitution was found of the third aspartate of this motif in Lbr_ChGH18 and GH18s of the most related species. GH18 chitinases break the glycosidic bonds of chitin through a substrate-assisted hydrolytic mechanism, in which the glutamate (E) acts as an acid/base residue and is essential for glycosidic bond cleavage, whereas the aspartate (D) residues present in these motifs primarily contribute to structural stabilization, substrate alignment, and maintaining the geometry of the active site [6]. Considering this, the Lbr_ChGH18 is expected to be enzymatically active. This is consistent with the observation in Lmx_ChGH18, a chitinase that also carries this substitution and is known to be active. Although this substitution may slightly alter the geometry of the catalytic site, it appears not to impair catalysis.

The importance for *Leishmania* to possess an active chitinase appears from previous reports showing that the intracellular survival of *L. major* depends on the constitutive internalization and degradation of host glycosaminoglycans, such as hyaluronan, by macrophages, which provide carbon sources, and create an exceptionally favorable niche for the parasite [77]. Hyaluronan is present and constitutively renewed in *Leishmania*-induced skin lesions and is internalized into phagolysosomes that contain the parasite, suggesting that it exploits important functions of macrophages.

Some reports have shown that the Lmx_ChGH18 acts as a multifunctional virulence factor that benefits the parasite throughout its life cycle. It even appears to facilitate the parasite's survival within macrophages [14]. Additionally, these chitinases in *Leishmania* have recently been suggested to be part of the secreted protein repertoire that mediates host–parasite interactions, potentially contributing to adaptation, virulence, and manipulation of the host or vector [78]. It is noteworthy that the chitinase of *Trichuris suis*, a helminth, directly interferes with dendritic cell (DC) activation and antigen presentation to CD4⁺ T cells [73]. This is relevant, considering the important role that DCs and CD4⁺ T cells play in the development of efficient anti-*Leishmania* immunity [79, 80]. Our subcellular localization predictions are consistent with these observations, indicating that several *Leishmania* chitinases are secreted into the extracellular environment, supporting their potential roles in modulating host–parasite interaction.

Domain sequence analyses of GH19 and GH20 chitinases revealed conservation of catalytic and substrate-binding residues, confirming enzymatic activity. These findings provide a first comprehensive map of GH18, GH19, and GH20 distribution in kinetoplastids, highlighting their need for evolutionary specialization of their chitinase repertoire.

RNA-seq analyses revealed a small but significant increase in the expression of the *LbrM.16.0800* gene in amastigotes compared with other stages, suggesting a stage-specific role. This is consistent with the expression data of *LbrM.16.0800* in the TriTrypDB database [81]—which provides Transcripts Per Million (TPM) and the Gene Expression Percentile (GEP). *LbrM.16.0800* reaches TPM values above 60 in amastigotes, indicating that the gene is expressed at high levels in amastigotes; its transcription is significantly more abundant than most genes with low or intermediate TPM. Similarly, the GEP value is above 70, indicating that *LbrM.16.0800* is expressed at levels higher than 70% of all genes in amastigotes and placing it among the group of genes with high relative (not just absolute) expression. This modest but significant increase in expression of this chitinase in amastigotes supports its potentially important role in the intracellular adaptation of *L. braziliensis*.

Lmex_ChGH18 mRNA is constitutively transcribed throughout the parasite’s life cycle, but with the expression of the protein significantly increased in amastigotes [14]. In all kinetoplastids analyzed, regulation of expression occurs primarily at the post-transcriptional level. The differences between *L. braziliensis* and *L. mexicana* described here may be due to differences in post-transcriptional regulation. This implies that, although the functional role of GH18 chitinases is likely conserved, the different species may employ distinct regulatory strategies to ensure appropriate enzyme availability dependent on the developmental stage or environmental conditions.

Structural predictions revealed that Lbr_ChGH18, as all GH18 enzymes, adopts the canonical TIM (β/α)_8_ barrel fold, with a deep substrate-binding cleft containing the catalytic residues [8, 82]. This analysis demonstrated that the catalytic site is precisely located within the barrel, as well as three SBM I, II, and III, which are positioned at the opposite ends of the groove, consistent with the classic GH18 arrangement [13]. This SBM plays a role in processivity and enzymatic activity toward crystalline chitin [13]. Structural superimposition with *Sm*ChiA revealed remarkable conservation of the overall fold and architecture of the catalytic site, highlighting the structural conservation across species. It is worth noting that Lbr_ChGH18 is structurally very similar to the chitinase of the bacterium *Micromonospora aurantiaca* (MaChi1), especially in a "tail" or flexible extension before the catalytic domain [83]. However, the possible function of this region, for example in protein stability or interaction with other proteins, is unknown.

In the docking analysis, the affinity values calculated by Vina ranged from −6.4 to −8.0 kcal/mol, which are considered moderate to strong. The substrate GlcNAc_6_ showed a slightly higher affinity for site II, which coincides with the SBM-I and SBM-II binding motifs involved in substrate stabilization and catalysis [14]. The difference in affinity between sites I and II could reflect the directionality of processing: site I could represent the initial binding position, with lower affinity, while site II offers greater stabilization, favoring substrate translocation during catalysis, similar to what has been reported in *Sm*ChiA [67, 84]. Using GlcNAc_6_ as a structural guide proved useful for identifying the SBMs and the potential pockets through which the substrate could pass. This allowed us to identify residues that could be important for substrate stabilization and movement along the groove. However, the conformational constraints of rigid docking do not capture the translocation dynamics of the substrate.

The case of closantel is particularly interesting: although it forms fewer direct contacts than argifin or argadin, it showed the strongest affinity at site II (−8.0 kcal/mol). This result indicates that its binding mode depends less on the total number of contacts and more on specific ones, probably related to the stabilization provided by residues present in the C-terminal region of the protein (R301–E305, D426–427, E429–433), which also participate in GlcNAc_6_ binding.

Unlike closantel, our analysis showed that both argifin and argadin, in addition to binding SBMs, also interact with catalytic residues, which is consistent with previous reports indicating that they inhibit enzyme activity by occupying the active site [57]. Furthermore, we also observed that their binding is based on a pattern of multiple interactions (such as hydrogen bonds, ionic contacts, hydrophobic interactions, and aromatic stacking), reflecting their nature as peptide mimetics adapted to carbohydrate-processing enzymes. These types of interactions were also observed when evaluating the inhibitory effect of both compounds on chitinase B from *S. marcescens* (*Sm*ChitB), especially the stacking interactions [57]. Together, these findings indicate that both compounds are likely to function as competitive inhibitors.

A key finding of these studies is the recurring involvement of the conserved motifs SBM-I, SBM-II, and SBM-III in the binding of all ligands evaluated. Residues such as H100, G101, and K103 (SBM-I); N149, E151, and Y152 (SBM-II); and D226, Y227, M231, Y233, D234, and H235 (SBM-III) consistently appear, forming critical contacts with the ligands. This highlights that these structural motifs represent essential elements for the arrangement of catalytic residues in the active site and substrate recognition. Consequently, targeted inhibition of these regions could have significant potential for modulating chitinase activity and the development of anti-*Leishmania* therapies.

Our immunoinformatic analyses of GH18 chitinases across clinically relevant *Leishmania* species reveal a pattern of conserved and subgenus-specific antigenic determinants. In New World species, several B-cell epitopes are highly conserved and often overlap predicted glycosylation motifs, suggesting that post-translational modifications may modulate epitope accessibility and immunogenicity. Subgenus-specific epitopes further indicate evolutionary diversification within Viannia and Leishmania (Leishmania), while Old World species retain a more conserved epitope repertoire. Importantly, several epitopes—including NLSGIMIWE, MSLMTAVHEQLA, and SPDVAE—are highly conserved between Old and New World species, suggesting potential targets for cross-species vaccine strategies. The predicted B-cell epitopes exhibit high antigenicity scores (BepiPred ≥ 0.55, ABCpred 0.68–0.95), supporting their potential to elicit humoral responses. None of the predicted *Leishmania* epitopes shares similarity with human chitinases, reinforcing the parasite-specific nature of these epitopes and reducing the likelihood of cross-reactivity.

T-cell epitope prediction revealed 10 high-confidence MHC-I nonamers with broad HLA coverage, including globally frequent alleles. Several peptides, binding multiple HLA-A and HLA-B molecules, could facilitate population-wide immunogenic responses.

Among these, only QTMTEWLNF (#9) is conserved between Old and New World species. This epitope is predicted to bind globally distributed human HLA molecules, including HLA-A and HLA-B alleles commonly found in populations across Asia, Africa, Europe, and the Americas [85, 86]. Other epitopes, such as SPMSLMAAV (3) and TVFGYLPEY (#8), exhibit minimal variation. Notably, SPMSLMAAV (#3) is predicted to bind to several HLA molecules; however, it primarily binds to HLA-B alleles, particularly those belonging to the B7 and B8 supertype families, which are prevalent in Africa, Asia, Europe, and the Americas [85]. The B8 supertype (HLA-B*08:01) is particularly frequent in Europe and North America [85], regions that are non-endemic but potentially exposed. Similarly, HLA-A*68:02 belongs to the A3 supertype, which is common in Africa [87], Latin America [88], and, to a lesser extent, Europe [85]. TVFGYLPEY (#8) may also be relevant because it binds to three of the most globally distributed HLA supertypes: A1 (HLA-A26:01 / HLA-A30:02), prevalent in Europe, North Africa, the Middle East, and West Africa; A3 (HLA-A11:01 / HLA-A68:01), found in Asia, Africa, and the Americas; and B7 (HLA-B35:01) [85, 89], present virtually worldwide. It is important to note that several of these alleles have been associated with slower progression and protection against multiple infectious diseases [89–92]. Additionally, it has been documented that some of these alleles may exert a protective effect against symptomatic manifestations of parasitic diseases [93, 94]. Given that QTMTEWLNF (#9), SPMSLMAAV (#3), and TVFGYLPEY (#8) bind to multiple globally distributed HLA molecules, they could be ideal candidates for constructing a multi-epitope. Such a construct could potentially generate immune responses in diverse populations by combining the global HLA coverage of each epitope into a single immunogen. This strategy not only maximizes population coverage but also streamlines vaccine design and may increase the likelihood of inducing robust cellular immunity against both Old and New World *Leishmania* species.

Similarly, 10 high-confidence MHC-II epitopes were predicted, each capable of binding multiple molecules of HLA-DPA/B, HLA-DQ, or HLA-DQA. Some peptides, such as MTEWLNFGRFLLELR (#5), TEWLNFGRFLLEIRS (#6), YEAFFKSGLTHLIFF (#7), and PHSSVEYAASVLSEE (#8), exhibit minimal variation and consistently bind the same HLA-II molecules. This suggests they contain core motifs compatible with HLA-DP binding. The shared HLA-binding profile, PB104:01 (frequent in Europe, the Americas, and Asia), DPB101:01 (frequent in Asia and the Americas), and DPB1*02:01 (frequent in Africa and the Americas), covers all regions where leishmaniasis is endemic, indicating that these epitopes may serve as universal targets for CD4^+^ T cells and support broad population coverage. HLA-DP alleles have been associated with protective factors for infectious diseases [95].

These analyses revealed the existence of three structurally conserved immunodominant regions within the chitinase protein. The convergence of overlapping peptides in recurrent sequence motifs (TEWLNFGRFLLER, HSSVEYAASVLSEE, and YEAFFK) suggests that these hotspots represent preferred recognition sites for CD4^+^ T lymphocytes. Their recognition by HLA-DP, DQ, and DR molecules would allow for greater population coverage in vaccine design. Importantly, the interaction of different MHC-II loci suggests that these hotspots may promote both early Th1 activation (associated with HLA-DP) [96], as well as sustained responses and even spontaneous clearance of infection (associated with HLA-DQ) [97], while maintaining their compatibility with several polymorphic HLA-DR alleles that predominate in human populations.

Taken together, these observations suggest that the identified hotspots could serve as essential universal auxiliary epitopes, providing long-lasting and widespread activation across genetically diverse populations and, potentially, across multiple *Leishmania* species.

Interestingly, partial overlaps were observed between predicted B-cell epitopes and both MHC-I and MHC-II peptides, indicating regions of potential dual immunogenicity. These overlapping regions may represent immunodominant sites capable of eliciting both humoral and cellular immune responses.

While several predicted epitopes are conserved between Old and New World *Leishmania* species, some peptides are group-specific. Although not conserved, they remain immunologically relevant, as they could Sinduce immune responses in populations exposed to *Leishmania* species of a given group. Including these species-specific epitopes in a multi-epitope vaccine could improve regional efficacy and broaden the overall response, complementing the coverage provided by the conserved epitopes.

Collectively, these findings provide a comprehensive immunoinformatic map of Lbr_ChGH18, highlighting conserved and group-specific antigenic determinants, the potential modulation of epitope accessibility mediated by glycosylation, and regions with dual immunogenic potential for B and T lymphocytes. This knowledge lays the groundwork for future experimental validation of therapeutic strategies that could reduce disease dissemination and the design of vaccines targeting multiple *Leishmania* species.

## Conclusions

This study provides a genomic analysis of kinetoplastid chitinases, revealing the presence of GH18, GH19, and GH20 chitinases, highlighting the diversity of gene repertoires and suggesting both evolutionary diversification and potential functional specialization, followed by a comprehensive characterization of GH18 from *L. braziliensis* (Lbr_ChGH18). The conservation of the catalytic motif indicates that Lbr_ChGH18 is likely enzymatically active, although it may also have functions beyond classical catabolism. Stage-specific expression analyses showed that gene *LbrM.16.0800* is highly expressed in all life-cycle stages but slightly, albeit significantly, more in amastigotes. Structural analyses confirmed the canonical TIM (β/α)_8_ barrel fold and the presence of substrate-binding motifs.

Docking studies onto Lbr_ChGH18 with GlcNAc_6_ and known chitinase inhibitors (closantel, argifin, and argadin) identified key residues involved in inhibitor recognition, providing insights into potential mechanisms of competitive inhibition. Additionally, identifying these inhibitory interaction sites may guide the future design of targeted therapeutic strategies for both human and veterinary applications, particularly considering the relevance of animal reservoirs in leishmaniasis transmission. Immunoinformatics analyses identified conserved B -and T -cell epitopes across Old and New World *Leishmania* species, capable of binding multiple HLA molecules. These epitopes exhibit broad population coverage and minimal similarity to human chitinases, supporting their potential as universal or region-specific vaccine candidates.

Taken together, our results integrate genomic, structural, functional, and immunological perspectives, establishing Lbr_ChGH18 as a model for understanding the evolution, function, and immunogenicity of chitinases, and laying the groundwork for future experimental validation, inhibitor design, and vaccine development against leishmaniasis.

## Electronic Supplementary Material

Below is the link to the electronic supplementary material.

Supplementary Material 1

Supplementary Material 2

## Data Availability

This article has no additional data.

## Abbreviations

GHs: Glycosyl hydrolases
GH18: Glycosyl hydrolases family 18
GH19: Glycosyl hydrolases family 19
GH20: Glycosyl hydrolases family 20
Lbr_ChGH18: *L. braziliensis* GH18 chitinase
GlcNAc: N-acetyl-D-glucosamine
NTD: Neglected tropical disease
CL: Cutaneous leishmaniasis
ML/MCL: Mucosal/mucocutaneous leishmaniasis
VL: Visceral leishmaniasis
NCBI: National Center for Biotechnology Information
CDD: Conserved domain database
SMART: Simple modular architecture research tool
GlcNAc6: Chitin hexamer
SBMs: Substrate-binding motifs
MHCI: Major histocompatibility complex I
MHCII: Major histocompatibility complex II
HLA: Human leukocyte antigens
RT_Nltr: Reverse transcriptase domain
CBD3: Chitin-binding
SH3b: Src homology 3 domain
ER: Endoplasmic reticulum
GA: Golgi apparatus
Lmex_ChGH18: *L. mexicana* GH18 chitinase
SP: Signal peptide
SBM I: Substrate-binding motif I
SBM II: Substrate-binding motif II
SBM III: Substrate-binding motif III
Chi-Kts: *Leishmania* Chitinase and related kinetoplastid
SmChiA: Chitinase A from *S. marcescens*
ORFs: Open-reading frames
pLbr_ChGH18: Processed Lbr_ChGH18
TPM: Transcripts per million
GEP: Gene expression percentile
MaChi1: *Micromonospora aurantiaca*
*Sm*ChitB: Chitinase B from *S. marcescens*
