# Supplementary material for: Characterization of GH18 chitinase in Leishmania braziliensis: expression, structural insights, and implications for vaccine and therapeutic development
Source: Biol Res. 2026 Apr 16;59:36. doi: 10.1186/s40659-026-00685-y (PMC13231604; doi:10.1186/s40659-026-00685-y)
Supplement: Supplementary file 1 — Supplementary Material 1 [file 40659_2026_685_MOESM1_ESM.docx]

**Supplementary Material**

**Supplementary Methodology**

**1. Phylogenetic Analysis of Chitinases**

The sequences used in a phylogenetic analysis correspond to representative chitinase-like proteins from a wide range of kinetoplastid species, including: *Leishmania major* (LmjF.16.0790), *Leishmania* *donovani* (LdBPK_160790.1), *Leishmania infantum* (LINF_160013400), *Leishmania tropica* (LTRL590_160013800), *Leishmania aethiopica* (LAEL147_000232000), *Leishmania braziliensis* (LbrM.16.0800), *Leishmania panamensis* (LPMP_160760), *Leishmania amazonensis* (LAMAPH8_000250000), *Leishmania guyanensis* (A0A1E1IUT6_LEIGU), *Leishmania mexicana* (LmxM.16.0790), *Leishmania shawi* (Q4I32_002489), *Leishmania utingensis* (Q4I30_002487), *Leishmania tarentolae Parrot* (LtaP16.0770), *Leishmania enriettii* (CUR178_05672), *Leishmania orientalis* (LSCM4_05615), *Leishmania* sp. Namibia (JIQ42_06049), *Leishmania* sp. Ghana (GH5_06168), *Porcisia hertigi* (JKF63_05287), *Endotrypanum monterogeii* (EMOLV88_160012400), *Leptomonas seymouri* (Lsey_0068_0030), *Leptomonas pyrrhocoris* (LpyrH10_15_0870), *Crithidia* *fasciculata* (CFAC1_120016000), *Angomonas deanei* (ADEAN_000766800), *Novymonas esmeraldas* (NESM_000322700), *Strigomonas culicis* (EPY29957.1), *Paratrypanosoma confusum* (PCON_0062580), *Blechomonas ayalai* (Baya_138_0020), *Trypanosoma melophagium*: *T. melophagium* 1 (LSM04_001139) and *T. melophagium* 2 (LSM04_001966), *Trypanosoma theileri* (TM35_000141060), *Bodo saltans*: *B. saltans* 1 (BSAL_89705) and *B. saltans* 2 (BSAL_28795), and *Trypanoplasma borreli* (GHOB01003007.1). In addition, from the diplonemids: *Diplonema papillatum*: *D. papillatum* 1 (KAJ9466790.1), *D. papillatum* 2.0 (KAJ9472914.1) and *D. papillatum* 2.1 (KAJ9449476.1); from the euglenids: *Euglena gracilis* (A0A2Z5U2B9_EUGGR), and from the heteroboloseans: *Naegleria gruberi*: *N. gruberi* 1(NAEGRDRAFT_62068), *N. gruberi* 2.0 (NAEGRDRAFT_68539), *N. gruberi* 2.1 (NAEGRDRAFT_81026), and *N. gruberi* 2.2 (NAEGRDRAFT_66142).

**2. RNA-seq data source**

Transcriptomic profiling of *L. braziliensis* throughout its life cycle was performed by reanalyzing publicly available RNA-seq datasets deposited in TriTrypDB [1]. These datasets correspond to three biological replicates for each of the three main developmental stages, totaling nine cDNA libraries, which were previously constructed and sequenced using Illumina next-generation sequencing (NGS) technology, generating approximately 677 million paired-end reads in total [2]. The raw sequencing reads were used to evaluate the expression profile of the *LbrM.16.0800* gene across developmental stages.

**Supplementary Tables**

**Supplementary Table 1. Chitinases and chitinase-related proteins identified by genomic analyses in different protists**

| **Organism** | **Number of chitinase genes** | **ID gene** | **Protein name** | **MW (kDa)** | **Associated domains** | **Length**  **a.a** | **Genomic localization** | **Subcellular localization** | **Database** |
| --- | --- | --- | --- | --- | --- | --- | --- | --- | --- |
| **Free-living** | | | | | | | | | |
| ***Naegleria*** | | | | | | | | | |
| *N. gruberi*  NEG-M | 5 | D2UZU7_NAEGR | Chitinase domain-containing protein 1 | 81.29 | 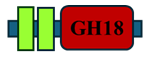 | 716 | Not Assigned | Mem Plas/**Perox** | Uniprot |
|  |  | D2VI28_NAEGR | Predicted protein | 21.49 | 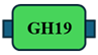 | 197 | Not Assigned | Extra |  |
|  |  | D2VS08_NAEGR | Predicted protein | 29.47 | 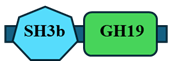 | 279 | Not Assigned | Extra |  |
|  |  | D2VBA0_NAEGR | Predicted protein | 29.65 | 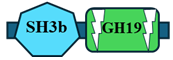 | 274 | Not Assigned | Extra/Lyso |  |
|  |  | D2VMQ6_NAEGR | Predicted protein | 44.08 | 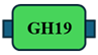 | 397 | Not Assigned | Extra |  |
| ***Euglena*** | | | | | | | | | |
| *E. gracilis* Z | 1 | A0A2Z5U2B9_EUGGR | Chitinase | 90.96 | 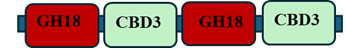 | 960 | Not Assigned | Extra | NCBI  Uniprot |
| ***Diplonema*** | | | | | | | | | |
| *D. papillatum*  ATCC50162 | 9 | KAJ9466790.1 | Chitinase | 40.10 | 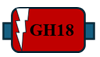 | 372 | Not Assigned | Extra | NCBI |
|  |  | KAJ9472914.1 | Chitinase | 38.67 | 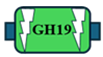 | 374 | Not Assigned | Extra |  |
|  |  | KAJ9467434.1 | Chitinase | 59.81 | 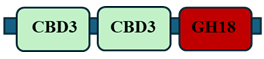 | 567 | Not Assigned | Extra/Lyso |  |
|  |  | KAJ9467433.1 | Chitinase | 104.41 | 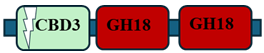 | 990 | Not Assigned | Extra/Lyso |  |
|  |  | KAJ9473400.1 | Chitinase domain-containing protein 1 | 45.14 | 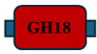 | 399 | Not Assigned | Extra/Lyso |  |
|  |  | KAJ9454922.1 | Gene 10 protein | 161.742 | 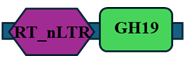 | 1529 | Not Assigned | **PM/Nucl/**  **Cyt/Mit** |  |
|  |  | KAJ9449476.1 | Endochitinase | 35.83 | 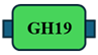 | 343 | Not Assigned | Extra |  |
|  |  | KAJ9449155.1 | Endochitinase | 35.85 | 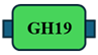 | 343 | Not Assigned | Extra |  |
|  |  | KAJ9449154.1 | Endochitinase | 35.85 | 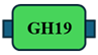 | 343 | Not Assigned | Extra |  |
| **Kinetoplastids** | | | | | | | | | |
| **Free-living** | | | | | | | | | |
| *Bodo saltans*  Lake Konstanz | 3 | BSAL_89705 | Glycoside hydrolase family 18, putative | 40.94 | 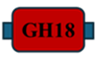 | 363 | Not Assigned | Extra/ER | TriTrypDB |
|  |  | BSAL_28795 | Glycoside hydrolase, putative | 32.27 | 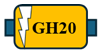 | 293 | Not Assigned | Extra/Cyt/Lyso |  |
|  |  | BSAL_68100 | Bacterial-type beta N-acetylhexosaminidase, putative | 89.21 | 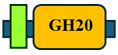 | 806 | Not Assigned | Cyt/Lyso/ER/  GA |  |
| **Obligate parasites** | | | | | | | | | |
| ***Cryptobia*** | | | | | | | | | |
| *Trypanoplasma borreli* strain Tt-JH | 1 | GHOB01003007 | TSA: Trypanoplasma borreli strain Tt-JH Tbor_DN712_c0_g1_i1, transcribed RNA sequence | 43.82 | 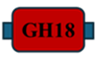 | 393 | Not Assigned | PM/Extra/ER | NCBI |
| ***Paratrypanosoma*** | | | | | | | | | |
| *P. confusum*  CUL13 | 1 | PCON_0062580 | Chitinase | 47.20 | 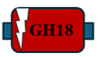 | 436 | Not Assigned | Extra/Lyso | TriTrypDB |
| ***Blechomonas*** | | | | | | | | | |
| *B. ayalai*  B08-376 | 1 | Baya_138_0020 | Chitinase | 51.95 | 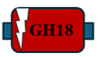 | 468 | Not Assigned | Extra/ER/GA | TriTrypDB |
| ***Trypanosoma*** | | | | | | | | | |
| *T. melophagium* St. Kilda | 8 | LSM04_001139 | Glycoside hydrolase family 20 | 59.55 | 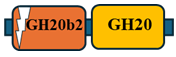 | 526 | Not Assigned | **PM**/Extra/Lyso | TriTrypDB |
|  |  | LSM04_003859 | Glycoside hydrolase family 20 | 59.55 | 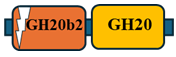 | 526 | Not Assigned | **PM**/Extra/Lyso | TriTrypDB |
|  |  | LSM04_008966 | Glycoside hydrolase family 20 | 37.88 | 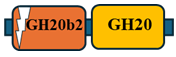 | 338 | Not Assigned | Extra | TriTrypDB |
|  |  | LSM04_008970 | Glycoside hydrolase family 20 | 59.55 | 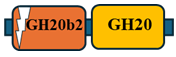 | 526 | Not Assigned | **PM**/Extra/Lyso | TriTrypDB |
|  |  | LSM04_004378 | Glycoside hydrolase family 20 | 59.55 | 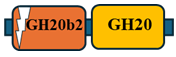 | 526 | Not Assigned | **PM**/Extra/Lyso | TriTrypDB |
|  |  | LSM04_002284 | Glycoside hydrolase family 20 | 59.55 | 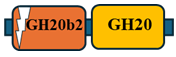 | 526 | Not Assigned |  | TriTrypDB |
|  |  | LSM04_008491 | Glycoside hydrolase family 20 | 59.55 | 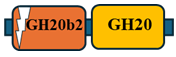 | 526 | Not Assigned | **PM**/Extra/Lyso | TriTrypDB |
|  |  | LSM04_007897 | Glycoside hydrolase family 20 | 59.55 | 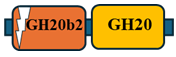 | 526 | Not Assigned | **PM**/Extra/Lyso | TriTrypDB |
| *T. theileri*  Edinburgh | 1 | TM35_000141060 | Beta-hexosaminidase subunit beta | 59.15 | 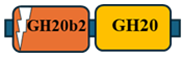 | 526 | Not Assigned |  | TriTrypDB |
| ***Leishmania*** | | | | | | | | | |
| *L. major*  Friedlin | 1 | LmjF.16.0790 | Chitinase | 50.55 | 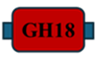 | 457 | 16 | Extra/ER/GA | TritTrypDB  Ensembl Protists |
| *L. donovani*  BPK282A1 | 1 | LdBPK_160790.1 | Chitinase | 50.45 | 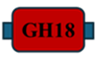 | 457 | 16 | Extra^*♦^/ER/GA | [3] TriTrypDB  Uniprot |
| *L. infantum* JPCM5 | 1 | LINF_160013400 | Chitinase | 50.39 | 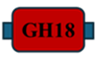 | 457 | 16 |  | TriTrypDB |
| *L. tropica* L590 | 1 | LTRL590_160013800 | Chitinase | 50.33 | 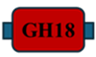 | 457 | 16 | ER/GA/Extra | TriTrypDB |
| *L. aethiopica* L147 | 1 | LAEL147_000232000 | Chitinase | 50.28 | 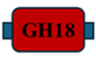 | 457 | 16 | ER/GA/Extra | TriTrypDB |
| **Viannia** | | | | | | | | | |
| *L. braziliensis*  MHOM/BR/75/M2904 | 1 | LbrM.16.0800 | Chitinase | 51.10 | 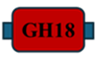 | 457 | 16 | Extra/ER/GA | TriTrypDB |
| *L. mexicana*  MHOM/GT/2001/U1103 | 1 | LmxM.16.0790 | Chitinase | 50.36 | 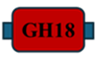 | 457 | 16 | Extra^*♦^/ER | [4]  TriTrypDB |
| *L. panamensis*  GCA 000755165.1 | 1 | LPMP_160760 | Chitinase-1 | 51.064 | 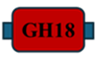 | 458 | 16 | Extra/ER/GA | TriTrypDB  Ensembl Protists  Uniprot |
| *L. guyanesi*  MHOM/BR/75/M4147/SSU:IR2SAT-LUC | 1 | A0A1E1IUT6_LEIGU | Putative chitinase | 51.11 | 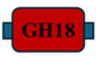 | 458 | --- | ER/Extra | UniProt |
| *L. amazonensis*  PH8 | 1 | LAMAPH8_000250000 | Glycosyl hydrolases family 18, putative | 50.66 | 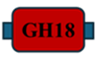 | 458 | 16 | ER/GA/Extra | TriTrypDB |
| **Sauroleishmania** | | | | | | | | | |
| *L. tarentolae*  Parrot-TarII | 1 | LtaP16.0770 | Chitinase | 50.80 | 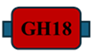 | 453 | 16 | Extra/ER/GA | TriTrypDB |
| **Mundinia** | | | | | | | | | |
| *L. enriettii* CUR178 | 1 | A0A836GIC9_LEIEN | GH18 domain-containing protein | 53.79 | 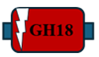 | 484 | 16 | Extra/Cyt/GA | Uniprot |
| ***Porcisia*** | | | | | | | | | |
| *P. hertigi*  MCOE/PA/1965/C119 | 1 | JKF63_05287 | Unspecified product | 50.94 | 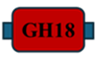 | 458 | 16 | Extra/ER/GA | TriTrypDB  Uniprot |
| ***Endotrypanum*** | | | | | | | | | |
| *E. monterogeii*  LV88 | 1 | EMOLV88_160012400 | Chitinase | 37.20 | 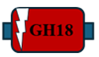 | 334 | 16 | **Mito/Nucl/**ER/Extra**/** | TriTrypDB |
| ***Leptomonas*** | | | | | | | | | |
| *L. seymouri*  ATCC 30220 | 1 | Lsey_0068_0030 | Chitinase | 50.43 | 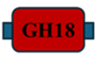 | 489 | Not Assigned | Extra/ER | Ensembl  Uniprot  TriTrypDB |
| *L. pyrrhocoris*  H10 | 1 | LpyrH10_15_0870 | Chitinase | 52.23 | 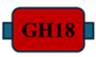 | 467 | 15 | Extra/ER | Ensembl Protists  Uniprot  TriTrypDB |
| ***Crithidia*** | | | | | | | | | |
| *C. fasciculata*  Cf-Cl | 1 | CFAC1_120016000 | Chitinase | 52.27 | 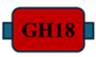 | 468 | 12 | Extra/ER | TriTrypDB |
| **Parasitic and host to an endosymbiotic bacterium** | | | | | | | | | |
| ***Angomonas*** | | | | | | | | | |
| *A. deanei*  Cavalho ATCC PRA-265 | 1 | ADEAN_000766800 | Glycosyl hydrolases family 18, putative | 41.67 | 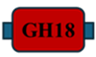 | 364 | 16 | Extra/ER | TriTrypDB |
| ***Novymonas*** | | | | | | | | | |
| *N. esmeraldas*  E262AT.01 | 1 | KAK7194099 | Chitinase | 48.99 | 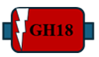 | 443 |  | Extra/ER | NCBI |
| ***Strigomonas*** | | | | | | | | | |
| *S. culicis*  GCA_000442495.1 | 2 | EPY29957.1 | Chitinase | 34.37 | 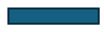 | 310 | Not Assigned | Extra | NCBI |
|  |  | EPY22137.1 | Chitinase | 58.54 | 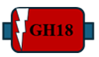 | 521 | Not Assigned | Extra/ER |  |

**Legend**

Chitinase sequences were retrieved from the National Center for Biotechnology Information (NCBI) database, Ensembl Genome database, the UniProt Consortium (UniProt), and the Kinetoplastid Genomic Resource (TriTrypDB). Domain and motif identification was performed using Protein BLAST (NCBI), the Conserved Domain Database (CDD), InterProScan (EMBL-EBI), and the Simple Modular Architecture Research Tool (SMART), with e-values ranging from 0.0 to 5 × 10⁻¹³, ensuring high-confidence detection. Subcellular localization annotations were based on either experimental evidence or predictions using computational tools, including WoLF PSORT, DeepLoc, and Cell-Ploc. Notation for subcellular localization: * predicted by bioinformatics analyses; ♦ experimentally validated. Localization symbology: Cytosol (Cyt); Nucleus (Nucl), Mitochondrion (Mitoch); Plasma membrane (PM); Lysosome (Lys); Extracellular (Extra); Golgi Apparatus (GA).

Glycoside Hydrolase Family 18 (GH18)
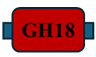
; GH18 domain having lost catalytic residues
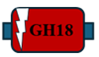
; Glycoside Hydrolase Family 19 (GH19)
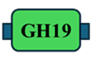
;

GH19 domain having lost chitin catalytic residues
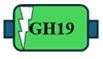
; GH19 domain having lost chitin binding and catalytic residues
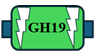
 ; Glycoside Hydrolase Family 20 (GH20)/β-hexosaminidase domain
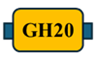
; GH20 domain having lost catalytic residues
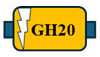
; β-hexosaminidase domain like having lost catalytic residues
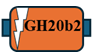
 ; Chitin-binding domain type 3
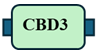
 ; Reverse transcriptase (RT) domain
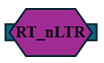
; Src homology 3 (SH3) -like domain (SH3b)
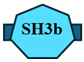
, Transmembrane domain
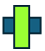
 ; Sequence without identified domain
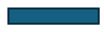
.

**Supplementary Table 2. Chitinases identified by genomic analyses in different isolates of *Leishmania* species**

| **Organism** | **Isolated** | **Number of chitinase gene** | **ID gene** | **Gene name** | **MW (kDa)** | **Associated domains** | **Length a.a** | **Genomic localization/ Chromosome** | **Subcellular localization** | **Database** |
| --- | --- | --- | --- | --- | --- | --- | --- | --- | --- | --- |
| ***Leishmania sensu stricto*** | | | | | | | | | |  |
| *L. major* | Friedlin 2021 | 1 | LMJFC_160014600 | Chitinase | 50.55 | 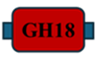 | 457 | 16 | ER/GA/Extra | TriTrypDB |
|  | LV39c5 | 1 | LMJLV39_160013600 | Chitinase | 50.67 | 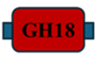 | 457 | 16 | ER/Extra | TriTrypDB |
|  | SD 75.1 | 1 | LMJSD75_160013400 | Chitinase | 50.55 | 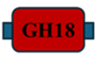 | 457 | Not Assigned | ER/GA/Extra | TriTrypDB |
| *L. donovani* | CL-SL | 1 | LdCL_160013200 | Chitinase | 50.45 | 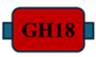 | 457 | 16 | ER/Extra | TriTrypDB |
|  | HU3 | 1 | LDHU3_16.0950 | Glyco 18 domain-containing protein | 50.45 | 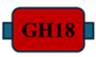 | 457 | 16 | ER/Extra | TriTrypDB |
|  | LV9 | 1 | LdBPK.16.2.000790 | Glycosyl hydrolases family 18, putative | 50.45 | 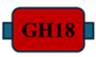 | 457 | 16 | ER/Extra | TriTrypDB |
| *L. infantum* | JPCM5 | 1 | LINF_160013400 | Chitinase | 50.39 | 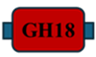 | 457 | 16 | ER/Extra | TriTrypDB |
| *L. amazonensis* | PH8 | 1 | LAMAPH8_000250000 | Glycosyl hydrolases family 18, putative | 50.66 | 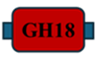 | 458 | 16 | ER/GA/Extra | TriTrypDB |
|  | MHOM/BR/71973/M2269 | 1 | LAMA_000249500 | Glycosyl hydrolases family 18, putative | 50.65 | 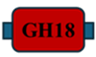 | 458 | Not Assigned | ER/Extra | TriTrypDB |
| ***Leishmania* viannia** | | | | | | | | | | |
| *L. braziliensis* | LbrM.16.2.000800 | 1 | LbrM.16.2.000800 | Glycosyl hydrolases family 18, putative | 51.01 | 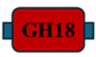 | 457 | 16 | ER/PM/Extra | TriTrypDB |
| *L. guyanesi* | IOCL 0565 | 1 | A0A6M8PIX0_LEIGU | Chitinase | 31.72 | 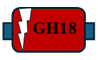 | 227 | --- | Cyt/Extra | UniProt |
| *L. shawi* | MHOM/BR/2013/18 LTA MLF | 1 | Q4I32_002489 | Glycosyl hydrolase family 18 | 51.10 | 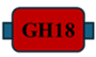 | 458 | Not Assigned | ER/Extra | UniProt |
| *L. utingensis* | ITUB/BR/1977/M4964 | 1 | Q4I30_002487 | Glycosyl hydrolase family 18 | 50.92 | 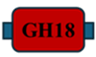 | 457 | Not Assigned | ER/Extra | UniProt |
| ***Sauroleishmania*** | | | | | | | | | | |
| *L. hertigi* | LEM452 | 1 | LGELEM452_160013100 | Chitinase |  | 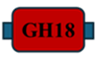 | 457 | 16 | ER/Extra |  |
| ***Mundinia*** | | | | | | | | | | |
| *L. orientalis* | LSCM4 | 1 | LSCM4_05615 | Unspecified product | 50.60 | 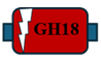 | 456 | Not Assigned | ER/PM/Extra | TriTrypDB |
| *L. martiniquensis* | LSCM1 | 1 | LSCM1_05715 | GH18 domain-containing protein | 50.67 | 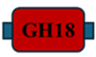 | 456 | Not Assigned | ER/Extra | UniProt  TriTrypDB |
| *L.* sp. Namibia | 253 | 1 | JIQ42_06049 | Unspecified product | 50.59 | 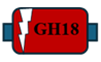 | 456 | 16 | ER/PM/Extra | UniProt  TriTrypDB |
| *L.* sp. Ghana 2012 LV757 | GH5 | 1 | GH5_06168 | GH18 domain-containing protein | 50.63 | 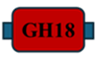 | 456 | 16 | ER/Extra | UniProt |

**Legend**

Glycoside Hydrolase Family 18 (GH18)
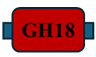
 ; GH18 domain having lost catalytic residues
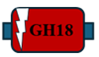
.

**Supplementary Table 3. E-values obtained for sequence similarity analysis of certain regions of chitinase enzymes described in this study with known canonical protein domains, using the servers and software specified below**

| **Organism** | **Accession**  **number** |  | | **e-value** | | | | | | |
| --- | --- | --- | --- | --- | --- | --- | --- | --- | --- | --- |
|  |  |  | | **Domains identified** | | | | | | |
|  |  | **GH18** | **GH19** | | **GH20** | **GH20b2** | **CBD3** | **SH3** | **CBD-A** | **RT-POL** |
| *N. gruberi*  *NEG-M* | D2UZU7_NAEGR | 2.89 e^-67^-1.50e^-23^ |  | |  |  |  |  |  |  |
|  | D2VI28_NAEGR |  | 2.51e-^78^ - 5.90e^-21^ | |  |  |  |  |  |  |
|  | D2VS08_NAEGR |  | 8.22 e^-78^ - 8.20^e-78^ | |  |  |  | 4.17e-^12^ - 1.32e^-04^ |  |  |
|  | D2VBA0_NAEGR |  | 6.58e-^76^ - 1.70e^-17^ | |  |  |  | 3.91e-07 - 2.35e-08 |  |  |
|  | D2VMQ6_NAEGR |  | 1.07e^-77^ -1.10e^-16^ | |  |  |  |  |  |  |
| *E. gracilis Z* |  |  | 2.27e-61 – 7.00e-06  3.84e-61- 2.00e-05 | |  |  |  |  | 1.24e^-11^ - 1.87e-^04^  1.96e^-11^ - 3.78e^-02^ |  |
| *D. papillatum*  ATCC50162 | KAJ9466790.1 | 4.60e-^41^ - 7.20e-^19^ |  | |  |  |  |  |  |  |
|  | KAJ9472914.1 | 2.40e-^63^ - 3.20e-^14^ |  | |  |  |  |  |  |  |
|  | KAJ9467434.1 | 6.95e-^117^- 9.26e-^104^ |  | |  |  | 8.21e-^15 -^1.15e^-10^ |  | 1.71e-^05^ -1.99e-^01^ |  |
|  | KAJ9467433.1 | 7.64e-^105^ – 1.85e-^92^  1.76e-^104^ - 6.48e-^92^ |  | |  |  | 7.15e-^11^-9.53e-^08^ |  |  |  |
|  | KAJ9473400.1 | 2.59e-^49^ - 7.89e-^05^ |  | |  |  |  |  |  |  |
|  | KAJ9454922.1 | 2.55e-^64^ - 4.60e-^19^ |  | |  |  |  |  |  | 9.64e-^32^-1.20e-^22^ |
|  | KAJ9449476.1 | 3.27e-^67^ - 3.50e-^15^ |  | |  |  |  |  |  |  |
|  | KAJ9449155.1 | 3.27e-^67^ - 3.50e-^15^ |  | |  |  |  |  |  |  |
|  | KAJ9449154.1 | 3.27e-^67^ - 3.50e-^15^ |  | |  |  |  |  |  |  |
| *B. saltans*  Lake Konstanz | BSAL_89705 | 1.65e-^42^ - 5.36e-^27^ |  | |  |  |  |  |  |  |
|  | BSAL_28795 |  |  | | 1.65e-^42^ - 1.50e-^22^ |  |  |  |  |  |
|  | BSAL_68100 |  |  | | 3.81e-78 - 7.00e^-63^ |  |  |  |  |  |
| *Trypanoplasma borreli* strain Tt-JH |  | 5.39e-^34^ - 3.49e-^17^ - |  | |  |  |  |  |  |  |
| *P. confusum*  CUL13 | PCON_0062580 | 3.06e-^242^ - 51e-^09^ |  | |  |  |  |  |  |  |
| *B. ayalai*  B08-376 | Baya_138_0020 | 1.10e-^27^ - 1.38e-^15^ |  | |  |  |  |  |  |  |
| *T. melophagium*  St. Kilda | LSM04_001139 |  |  | | 5.30e-^135^-4.50e-^79^ | 1.44e-^11^ -1.70e-^10^ |  |  |  |  |
|  | LSM04_003859 |  |  | | 5.30e-^135^ - 4.50e-^79^ | 1.44e-^11^ -1.70e-^10^ |  |  |  |  |
|  | LSM04_008966 |  |  | | 8.18e-^40^ - 2.10e-^25^ | 4.60e-^11^ -4.00e-^06^ |  |  |  |  |
|  | LSM04_008970 |  |  | | 5.30e-^135^ - 4.50e-^79^ | 1.44e-^11^ -1.70e-^10^ |  |  |  |  |
|  | LSM04_004378 |  |  | | 5.30e-^135^ - 4.50e-^79^ | 1.44e-^11^ -1.70e-^10^ |  |  |  |  |
|  | LSM04_002284 |  |  | | 5.30e-^135^ - 4.50e-^79^ | 1.44e-^11^ -1.70e-^10^ |  |  |  |  |
|  | LSM04_008491 |  |  | | 5.30e-^135^ - 4.50e-^79^ | 1.44e-^11^ - 1.70e-^10^ |  |  |  |  |
| *T. theileri*  Edinburgh | TM35_000141060 |  |  | | 1.93e-139 - 2.40e-^82^ | 2.80e-^14^ - 5.75e-^13^ |  |  |  |  |
| *L. major* Friedlin | LmjF.16.0790 | 4.60e-^41^ - 3.59e-^28^ |  | |  |  |  |  |  |  |
| *L. donovani*  BPK282A1 | LdBPK_160790.1 | 7.56e-^44^ - 5.40e-^32^ |  | |  |  |  |  |  |  |
| *L. infantum* JPCM5 | LINF_160013400 | 1.19e-^43^ - 1.06e-^30^ |  | |  |  |  |  |  |  |
| *L. tropica* L590 | LTRL590_160013800 | 7.08e-^27^ - 1.31e-^40^ |  | |  |  |  |  |  |  |
| *L. aethiopica* L147 | LAEL147_000232000 | 8.56e-^41^ - 1.23e-^26^ |  | |  |  |  |  |  |  |
| *L. braziliensis*  MHOM/BR/75/M2904 | LbrM.16.0800 | 1.84e-^33^ - 3.77e-^22^ |  | |  |  |  |  |  |  |
| *L. mexicana*  MHOM/GT/2001/U1103 | LmxM.16.0790 | 3.82e-^41^ - 6.35e-^29^ |  | |  |  |  |  |  |  |
| *L. panamensis*  GCA 000755165.1 | LPMP_160760 | 5.80e-^23^ - 5.74e-^33^ |  | |  |  |  |  |  |  |
| *L. guyanesi*  MHOM/BR/75/M4147/  SSU:IR2SAT-LUC | A0A1E1IUT6_LEIGU | 2.53e-^33^ - 7.77e-^24^ |  | |  |  |  |  |  |  |
| *L. amazonensis*  PH8 | LAMAPH8_000250000 | 2.34e-^41^ - 3.25e-^26^ |  | |  |  |  |  |  |  |
| *L. tarentolae*  Parrot-TarII | LtaP16.0770 | 3.51e-^45^- 3.66e-^28^ |  | |  |  |  |  |  |  |
| *L. enriettii* CUR178 | CUR178_05672 | 4.35e-^44^ -1.97e-^25^ |  | |  |  |  |  |  |  |
| *Porcisia hertigi*  MCOE/PA/1965/C119 | JKF63_05287 | 4.38 e-^4^ -1.24e-^22^ |  | |  |  |  |  |  |  |
| *E. monterogeii*  LV88 | EMOLV88_160012400 | 3.60e-^40^- 3.24e^35^ |  | |  |  |  |  |  |  |
| *L. seymouri*  ATCC 30220 | Lsey_0068_0030 | 6.95e^-43^ - 9.34e^-27^ |  | |  |  |  |  |  |  |
| *L. pyrrhocoris*  H10 | LpyrH10_15_0870 | 5.61e^-46^ -5.53e^-29^ |  | |  |  |  |  |  |  |
| *C. fasciculata*  Cf-Cl | CFAC1_120016000 | 3.77e^-45^ - 5.00e-^27^ |  | |  |  |  |  |  |  |
| *A. deanei*  Cavalho ATCC PRA-265 | ADEAN_000766800 | 6.99e^-29^ - 1.11e^-06^ |  | |  |  |  |  |  |  |
| *N. esmeraldas*  E262AT.01 | KAK7194099 | 1.78e-31 - 1.47e^-24^ |  | |  |  |  |  |  |  |
| *S. culicis*  GCA_000442495.1 | EPY22137.1 | 2.68e^-16^ - 7.21e^-10^ |  | |  |  |  |  |  |  |

**Legend**

E-values were obtained from the sequence similarity analysis of chitinase catalytic regions and associated domains. Conserved domains were identified using the Conserved Domain Database (CDD), InterProScan (EMBL-EBI), and the Simple Modular Architecture Research Tool (SMART). The analysis enabled the identification of GH18, GH19, and GH20 catalytic domains, along with additional associated modules when present. E-values represent the degree of similarity between predicted regions and canonical domain profiles in each database.

**Supplementary Table 4. Refinement statistics and stereochemical quality of Lbr_ChGH18 models generated by GalaxyWeb.**

| **Model** | **GDT-HA** | **RMSD** | **MolProbity** | **Clash score** | **Poor rotamers** | **Rama favored** |
| --- | --- | --- | --- | --- | --- | --- |
| Initial | 1.0000 | 0.000 | 1.425 | 7.8 | 0.8 | 98.9 |
| MODEL 1 | 0.9819 | 0.263 | 1.498 | 9.4 | 0.5 | 98.9 |
| **MODEL 2** | **0.9902** | **0.222** | **1.504** | **9.6** | **0.3** | **98.9** |
| MODEL 3 | 0.9885 | 0.226 | 1.525 | 10.1 | 0.3 | 98.9 |
| MODEL 4 | 0.9896 | 0.225 | 1.565 | 11.2 | 0.3 | 98.9 |
| MODEL 5 | 0.9907 | 0.229 | 1.504 | 9.6 | 0.3 | 98.7 |

**Supplementary Table 5. Refinement statistics and stereochemical quality of pLbr_ChGH18 models generated by GalaxyWeb.**

| **Model** | **GDT-HA** | **RMSD** | **MolProbity** | **Clash score** | **Poor rotamers** | **Rama favored** |
| --- | --- | --- | --- | --- | --- | --- |
| Initial | 1.0000 | 0.000 | 1.452 | 8.3 | 0.8 | 98.6 |
| **MODEL 1** | **0.9659** | **0.371** | **1.471** | **8.8** | **0.0** | **98.8** |
| MODEL 2 | 0.9700 | 0.331 | 1.484 | 9.1 | 0.3 | 98.6 |
| MODEL 3 | 0.9700 | 0.335 | 1.530 | 10.2 | 0.3 | 98.8 |
| MODEL 4 | 0.9665 | 0.344 | 1.439 | 8.1 | 0.6 | 98.6 |
| MODEL 5 | 0.9815 | 0.294 | 1.541 | 10.5 | 0.0 | 98.4 |

**Legend for Tables 4 and 5**

The tables summarize key validation metrics for the initial and five refined models.

The model highlighted in bold was the refined model chosen.

Rama favored: Percentage of residues in favored regions of the Ramachandran plot; values ≥98% indicate excellent backbone geometry.

**Supplementary Table 6. Predicted linear B-cell epitopes and potential glycosylation sites in New World *Leishmania* chitinases identified using multiple computational tools**

| **Species** | **ID Gene** | **BepiPred 2.0** | | **ABCpred** |  | **GlycoEP/NetNGlyc** | |
| --- | --- | --- | --- | --- | --- | --- | --- |
|  |  | **Sequence** | **Score** | **Sequence** | **Score** | **sequons** | **Score** |
| *L. braziliensis*  MHOM/BR/75/M2904 | LbrM.16.0800 | VAAAASAAVRDSAARSSSQNASIT | 0.55 | **AAAASAAVRDSAARSS** | 0.68 | N*ASI | 0.61-1.00 |
|  |  | GGGGRSSGFSDL | 0.55 | **GGGGRSSGFSDL**VGDP | 0.86 |  |  |
|  |  | MALHPHSS | 0.55 | AVLS**MALHPHSS**IAAV | 0.71 |  |  |
|  |  | TYNKRSGQTHQEL | 0.55 | **KRSGQTHQEL**RTEQDH | 0.86 |  |  |
|  |  | NLSGIMIWEL **^#^** | 0.55 | **GIMIWEL**GQDVPPGTS | 0.89 | N*LSG | 0.65-0.93 |
|  |  | PMSLMAAVHEQLAD **^#^** | 0.55 | VPPGTSP**MSLMAAVHE** | 0.77 |  |  |
|  |  | TDSGHDSGGDVNGDN | 0.55 | **TDSGHDSGGDVNGDN**T | 0.90 |  |  |
|  |  | RQRRYLSSDVAED **^#^** | 0.55 | QQ**RQRRYLSSDVAED**G | 0.65 |  |  |
|  |  |  |  |  |  |  |  |
| *L. mexicana*  MHOM/GT/  2001/U1103 | LmxM.16.0790 | RSSLVQL |  | **--------** |  |  |  |
|  |  | DTAIGSGHNTSIT | 0.55 | **TAIGSGHNTSIT**AASP | 0.89 | N*TSI | 0.65-0.72 |
|  |  | GAQLMLCIGGGGRSAG | 0.55 | **MLCIGGGGRSA**GFADL | 0.77 |  |  |
|  |  | SLGYAAAAGD | 0.55 | S**SLGYAAAAGD**EHAGK | 0.88 |  |  |
|  |  | KNHPTWV | 0.55 | EWAS**KNHPTWV**EGGAE | 0.87 |  |  |
|  |  | NLSGIMIWE **^#^** | 0.55 | MRLARAA**NLSGIMIWE** | 0.80 | N*LSG | 0.65-0.93 |
|  |  | MSLMTAVHEQLAD **^#^** | 0.55 | L**MTAVHEQLAD**WGLLT | 0.70 |  |  |
|  |  | TDSRHGSGGNVNSGNA | 0.55 | **HGSGGNVNSGNA**YDCP | 0.95 |  |  |
|  |  | SPDVAE **^#^** | 0.55 | QPLPQDS**SPDVAE**DRD | 0.76 |  |  |
|  |  |  |  |  |  |  |  |
| *L. amazonensis*  MHOM/BR/71973/M2269 | LAMAPH8_000250000 | RSSLVQL |  | **---------** | ----- |  |  |
|  |  | AAAASAAAGDTAISSRHNTSIAE | 0.55 | **TAISSRHNTSIAEASP** | 0.93 | N*TSI | 0.65-0.73 |
|  |  | GAQLMLCIGGGGRSAG | 0.55 | **MLCIGGGGRSAG**FADL | 0.77 |  |  |
|  |  | RSSLGYSAAAGD | 0.55 | **SSLGYSAAAGD**EHAGK | 0.85 |  |  |
|  |  | SVLSEGT | 0.55 | SVEYAA**SVLSEGT**IGL | 0.67 |  |  |
|  |  | KNHPTWV | 0.55 | EWAS**KNHPTWV**EGGAE | 0.87 |  |  |
|  |  | NLSGIMIWE **^#^** | 0.55 | MRLARAA**NLSGIMIWE** | 0.80 | N*LSG | 0.65-0.93 |
|  |  | MSLMTAVHEQLAD **^#^** | 0.55 | **LMTAVHEQLAD**WGLLT | 0.70 |  |  |
|  |  | DSRHGSGGNVNSGNA | 0.55 | **HGSGGNVNSGNA**YDCP | 0.95 |  |  |
|  |  | SPDVAE **^#^** | 0.55 | QPLPQDS**SPDVAE**DRD | 0.76 |  |  |
|  |  |  |  |  |  |  |  |
| *L. panamensis*  GCA 000755165.1 | LPMP_160760 | VAAAASAAVRDSAASSSSSQNASIT | 0.55 | **ASSSSSQNASIT**TASP | 0.80 | N*ASI | 0.60-1.17 |
|  |  | GGGGRSSGFSD ° | 0.55 | **GGGGRSSGFSD**LVGDP | 0.86 |  |  |
|  |  | SMALHPHSS | 0.55 | AVL**SMALHPHSS**IAAV | 0.71 |  |  |
|  |  | TYNKRSGQ | 0.55 | FSEA**TYNKRSGQ**TRQE | 0.80 |  |  |
|  |  | NLSGIMIWEL**^#^** | 0.55 | MRLARAA**NLSGIMIWE** | 0.80 | N*LSG | 0.65-0.93 |
|  |  | PMSLMAAVHEQLAD **^#^** | 0.55 | VPPGTS**PMSLMAAVHE** | 0.77 |  |  |
|  |  | LLTDSGHGSGGDVNGD | 0.55 | **HGSGGDVNGD**DTGHLQ | 0.86 |  |  |
|  |  | SPDVAEDG **^#^** | 0.55 | QQRQRRYL**SPDVAE**DG | 0.76 |  |  |
|  |  |  |  |  |  |  |  |
| *L. guyanensi*  IOCL 0565 | A0A6M8PIX0_LEIGU | VAAAESAAVRDSAASSSSSQNASIT | 0.55 | **ASSSSSQNASIT**TASP | 0.80 | N*ASI | 0.60-1.17 |
|  |  | GGGGRSSGFSD | 0.55 | **GGGGRSSGFSD**LVGDP | 0.86 |  |  |
|  |  | SMALHPHS | 0.55 | AVL**SMALHPHS**SIAAV | 0.71 |  |  |
|  |  | ATYNKRSGQTHQELR | 0.55 | **KRSGQTHQELR**TEQDH | 0.86 |  |  |
|  |  | NLSGIMIWEL **^#^** | 0.55 | MRLARAA**NLSGIMIWE** | 0.80 | N*LSG | 0.65-0.93 |
|  |  | PMSLMAAVHEQLAD **^#^** | 0.55 | **EQLAD**WGLLTDSGHGS | 0.81 |  |  |
|  |  | LTDSGHGSGGDVNGD | 0.55 | **HGSGGDVNGDD**TGHLQ | 0.86 |  |  |
|  |  | SPDVAEDG ^#^ | 0.55 | QQRQRRYL**SPDVAEDG** | 0.76 |  |  |

**Legend**

Epitope prediction by BepiPred 2.0 was performed considering both amino-acid sequence and structural features, with a threshold score of 0.55, while ABCpred employed an artificial neural network–based algorithm with a cutoff score >0.50. Glycosylation site prediction was conducted using two independent computational tools, both applying a threshold value of 0.50 to identify potential N- and O-linked glycosylation motifs within the same protein regions. For each *Leishmania* species and gene identifier, the table summarizes the predicted epitope sequence, glycosylation status, and corresponding prediction scores. N* denotes an asparagine residue predicted to be glycosylated. Bold letters indicate common amino-acid sequences shared between epitopes predicted by BepiPred 2.0 and ABCpred. # Epitopes also preserved in all *Leishmania* species of the New World.

**Supplementary Table 7. Predicted linear B-cell epitopes and potential glycosylation sites in Old World *Leishmania* chitinases identified using multiple computational tools**

| **Species** | **ID Gene** | **BepiPred 2.0** | | **ABCpred** | |  | **GlycoEP/****NetNGlyc** | |
| --- | --- | --- | --- | --- | --- | --- | --- | --- |
|  |  | **Sequence** | **Score** | **Sequence** | **Score** | | **sequons** | **Score** |
| *L. major*  Friedlin | LmjF.16.0790 | TAISSRHNTSVTA | 0.55 | **TAISSRHNTSVTA**ASP | 0.81 | | **N***TSV | 0.64-0.77 |
|  |  | SLGYAAA | 0.55 | **SSLGYAAA**AGGEHAAE | 0.80 | |  |  |
|  |  | QSARML | 0.55 | HPSMAAVL**QSARML**HS | 0.72 | |  |  |
|  |  | KNHPTW | 0.55 | EWASK**NHPTWVE**GGAE | 0.87 | |  |  |
|  |  | LSGIMIW **^#^** | 0.55 | MRLARAAN**LSGIMIW** | 0.80 | | **N***LS | 0.75-0.93 |
|  |  | MSLMTAVHEQLAD **^#^** | 0.55 | **EQLAD**WGLLTDSGRGI | 0.86 | |  |  |
|  |  | GLLTDSGRGIGGNVNSDNA º | 0.55 | **RGIGGNVNSDNA**HDRP | 0.81 | |  |  |
|  |  | SPDVAE **^#^** | 0.55 | DRPQPSPQH**SSPDVAE** | 0.81 | |  |  |
|  |  |  |  |  |  | |  |  |
| *L. tropica* | LTRL590_160013800 | AISSRHNTSIT | 0.55 | **TAISSRHNTSITAA**SP | 0.84 | | N*TSI | 0.71-0.69 |
|  |  | KNHPT | 0.55 | EWAS**KNHPTW**VEGGAE | 0.87 | |  |  |
|  |  | LSGIMI **^#^** | 0.55 | MRLARAAN**LSGIMI**WE | 0.80 | | N*LSG | 0.64-0.94 |
|  |  | MSLMTAVHEQLA**^#^** | 0.55 | **EQLAD**WGLLNDSGRGS | 0.99 | |  |  |
|  |  | GLLNDSGRGSGGNVKSDNAº | 0.55 | **SGRGSGGNVKSDNA**CD | 0.85 | | N*DSG | 0.60 -0.99 |
|  |  | PDVAE **^#^** | 0.55 | CDRPQPPPRH**SSPDVA** | 0.88 | |  |  |
|  |  |  |  |  |  | |  |  |
| *L. aethiopica* | LAEL147_000232000 | TAISSRHNTSITAASS | 0.55 | **TAISSRHNTSITAASS** | 0.76 | | N*TSI | 0.72-0.67 |
|  |  | SLGYAAAAGG | 0.55 | S**SLGYAAAAG**GEHAAG | 0.72 | |  |  |
|  |  | NHPTW | 0.55 | EWASK**NHPTWV**EGGAE | 0.87 | |  |  |
|  |  | NLSGIMIWE **^#^** | 0.55 | MRLARAA**NLSGIMIWE** | 0.80 | | N*LSG | 0.70-0.93 |
|  |  | MSLMTAVHEQLA **^#^** | 0.55 | GQDVPPGTSP**MSLMTA** | 0.75 | |  |  |
|  |  | GLLTDSGRGSGGNVKSGNA | 0.55 | **SGRGSGGNVKSGNA**CD | 0.87 | |  |  |
|  |  | PDVAED **^#^** | 0.55 | QPPPQHSS**PDVAE**DGD | 0.76 | |  |  |
|  |  |  |  |  |  | |  |  |
| *L. donovani*  BPK282A1 | LdBPK_160790.1 | TAISSRHNTPITAA | 0.55 | **TAISSRHNTPITAA**SP | 0.89 | |  |  |
|  |  | RSSLGYAAAAGGEHA | 0.55 | **SSLGYAAAAGGEHA**AG | 0.72 | |  |  |
|  |  | KNHPTWV | 0.55 | EWASK**NHPTWV**EGGAE | 0.87 | |  |  |
|  |  | RAANLSGIMIWEL **^#^** | 0.55 | **GIMIWEL**GQDVPPGTS | 0.82 | | N*LSG | 0.68-0.98 |
|  |  | MSLMTAVHEQLAD **^#^** | 0.55 | **LMTAVHEQLAD**WGLLT | 0.70 | |  |  |
|  |  | GLLTDGGRGSGGNVNSDNA | 0.55 | **GLLTDGGRGSGGN**VNS | 0.81 | |  |  |
|  |  | SSPDVAED **^#^** | 0.55 | QPPPQH**SSPDVAED**GD | 0.76 | |  |  |
|  |  |  |  |  |  | |  |  |
| *L. infantum* JPCM5 | LINF_160013400 | TAISSRHNTPI | 0.55 | **TAISSRHNTPI**TVASP | 0.93 | |  |  |
|  |  | RSSLGYAAAAGGEHA | 0.55 | **SSLGYAAAAGGEHA**AG | 0.72 | |  |  |
|  |  | SHPTWVE | 0.55 | EWASK**SHPTWVE**GGAE | 0.88 | |  |  |
|  |  | TRAANLSGIMIWEL **^#^** | 0.55 | **GIMIWEL**GQDVPPGTS | 0.88 | | N*LSG | 0.68-0.98 |
|  |  | MSLMTAVHEQLAD **^#^** | 0.55 | GQDVPPGTSP**MSLMTA** | 0.75 | |  |  |
|  |  | GLLTDGGRGSGGNVNSDNA º | 0.55 | **GLLTDGGRGSGGNVNS** | 0.81 | |  |  |
|  |  | SPDVAED**^#^** | 0.55 | QPPPQHSS**PDVAE**DGD | 0.90 | |  |  |
|  |  |  |  |  |  | |  |  |

**Legend**

Epitope prediction using BepiPred 2.0 was performed based on both amino-acid sequence and structural features, with a threshold score of 0.55. At the same time, ABCpred employed an artificial neural network–based algorithm with a cutoff score >0.50. Glycosylation site prediction was conducted using two independent computational tools, both applying a threshold value of 0.50 to identify potential N- and O-linked glycosylation motifs within the same protein regions. For each *Leishmania* species and gene identifier, the table summarizes the predicted epitope sequence, glycosylation status, and corresponding prediction scores. N* denotes an asparagine residue predicted to be glycosylated. Bold letters indicate common amino-acid sequences shared between epitopes predicted by BepiPred 2.0 and ABCpred. **^#^**  Epitopes also preserved in New World *Leishmania* species.

**Supplementary Table 8. Comparative amino-acid sequence identity of *Leishmania* and human chitinases**

**
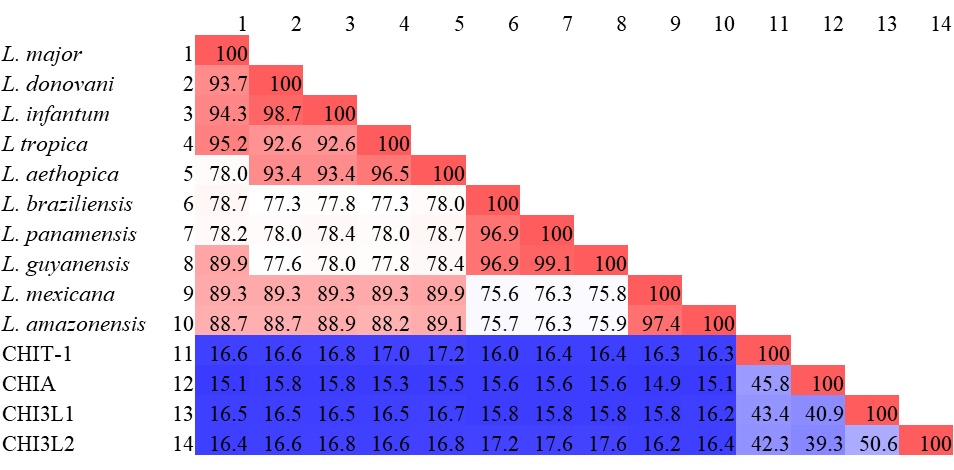
**

**Legend**

Percentages of pairwise identities of sequences were calculated from their multiple/pairwise alignment using CLC Main Workbench 25.0.3 (Qiagen). The percentage identity is shown on a color scale ranging from blue (lower values) to red (higher values). Human chitinases: Chitotriosidase-1 (CHIT-1); Acidic mammalian chitinase (CHIA); Chitinase-3-like protein 1 (CHI3L1); Chitinase-3-like protein 2 (CHI3L2).


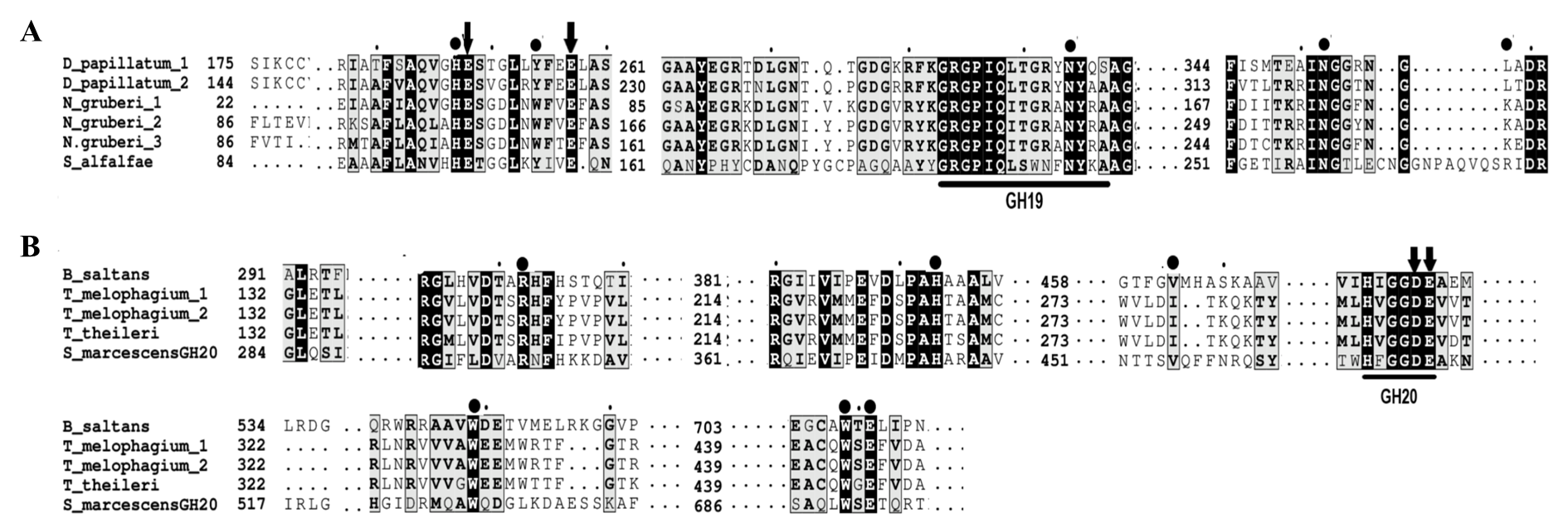


**Supplementary Figure 1. Partial amino-acid sequence alignment of chitinase domains**. Alignment was performed with the program T-COFFEE [5] (**A) Sequence alignment of chitinase GH19 domains.** The underlined sequence indicates residues of the chitinase-19 signature. The putative catalytic sites are indicated by down-pointing arrows at the top, and the substrate binding sites with black circles. Sequences from GH19 chitinases are shown as follows: *Diplonema papillatum: D. papillatum* 1 (KAJ9472914.1), *D. papillatum 2* (KAJ9449476.1); *Naegleria. gruberi:* N. gruberi_1 (NAEGRDRAFT_68539), N. gruberi_ 2 *(*NAEGRDRAFT_81026), N. gruberi _3 (NAEGRDRAFT_66142), and *Streptomyces alfalfae* (RZM91334.1**). B) Sequence alignment of chitinase GH20 domains.** Residues involved in chitin binding are indicated with black circles. Residues involved in the catalytic/active site are indicated by down-pointing arrows at the top. Sequences are shown as follows: *Bodo saltans* (B_saltans_68100), *Trypanosoma theileri (*TM35_000141060), *Trypanosoma melophagium*: T_melophagium_1 (LSM04_003859), T_melophagium_2 (LSM04_008966), and *Serratia marcescens* GH20 (WP_055316095). Black boxes indicate amino-acid residues that, in all sequences, are identical. Well conserved amino-acid residues are indicated by gray backgrounds. White boxes indicate residue positions where only conservative substitutions are found, and dots indicate the absence of amino acids at corresponding positions.

.

**Supplementary Figures**

**
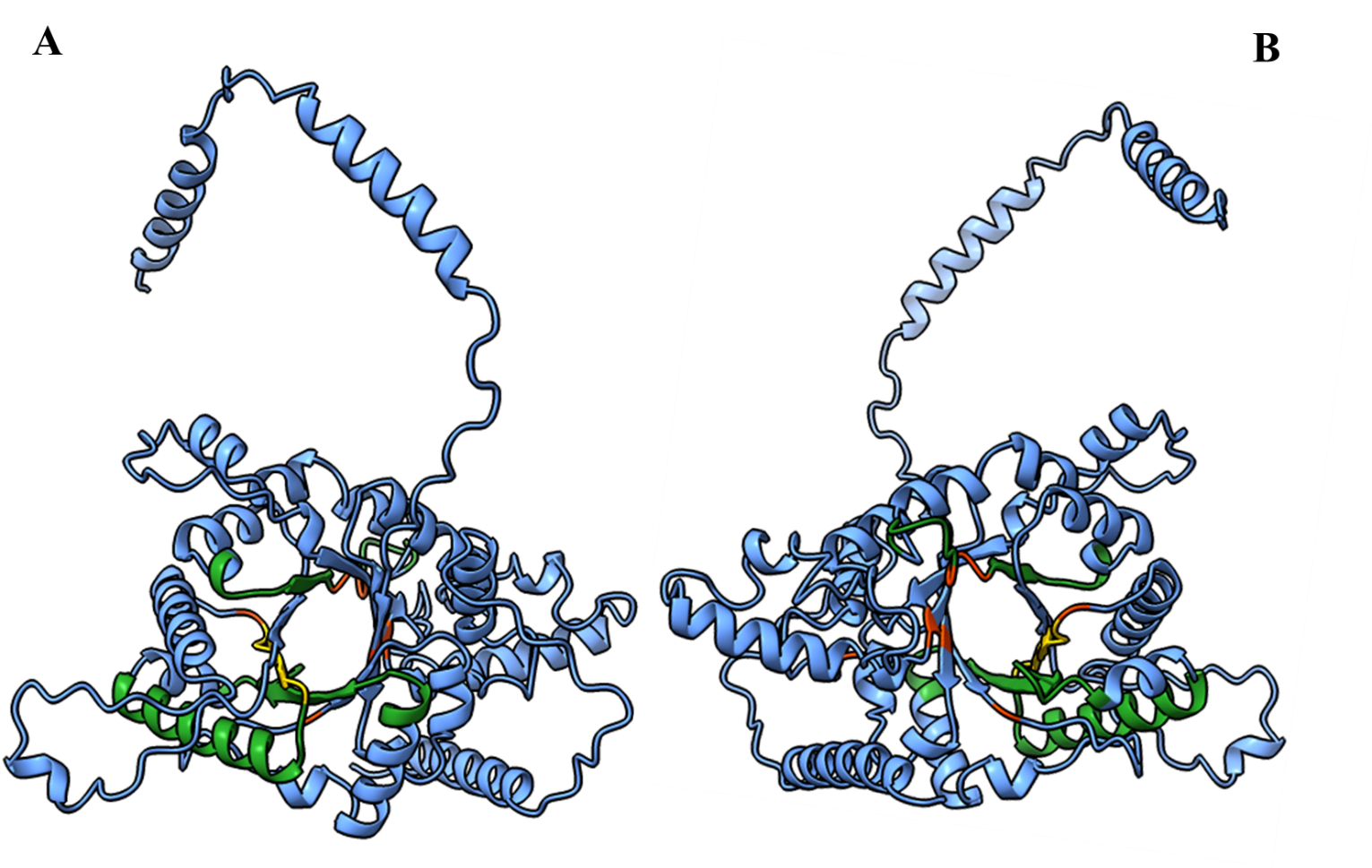
**

**Supplementary Figure 2. Structure modeling and refinement of GH18 chitinases from *L. braziliensis* (Lbr_ChGH18)*.*** Front (**A**) and rear (**B**) views of the full-length Lb_ChGH18 showing the canonical chitinase fold with a TIM-barrel (β/α)₈ domain characteristic of GH18 enzymes. Highlighted parts correspond to the residues of conserved DXDXXDXE catalytic motif (yellow), residues forming the chitinase domain (red), and substrate-binding motif (SBM I-III) (green).


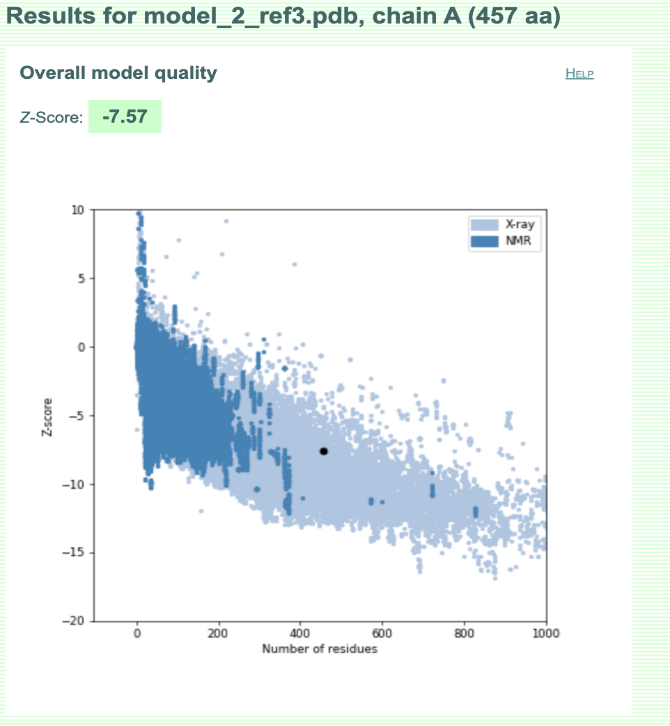

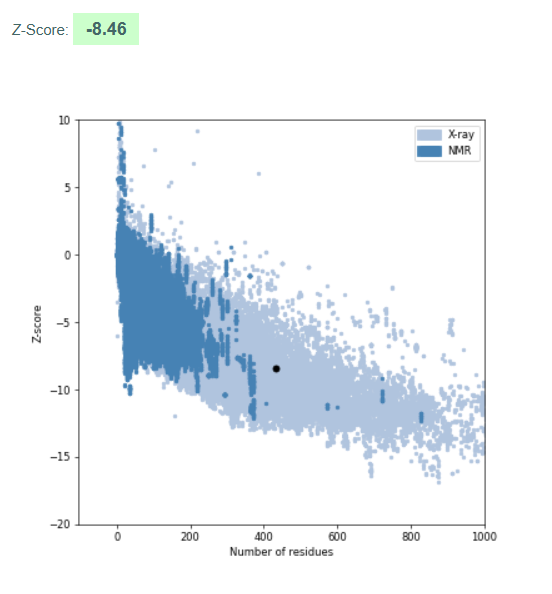


**A**

**B**

**Supplementary Figure 3. Validation of refined Lbr_ChGH18 structural models using ProSA.**

(**A**) Full-length chitinase model (Lbr_ChGH18) showing a Z-score of −7.57, consistent with values typically observed for experimentally determined proteins of similar size. (**B**) Processed chitinase model (pLbr_ChGH18), created after removal of the signal peptide, displaying an improved Z-score of −8.40, indicating higher structural stability and reliability. Both models fall within the range of high-quality protein structures, supporting the overall accuracy of the predicted folds.


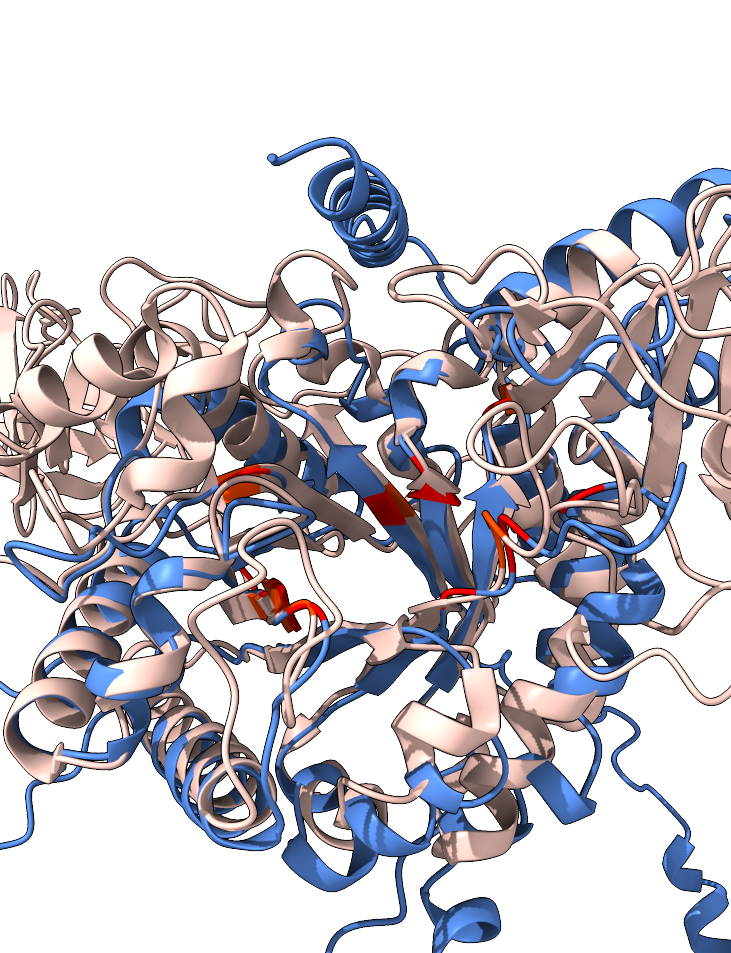


**B**

**A**


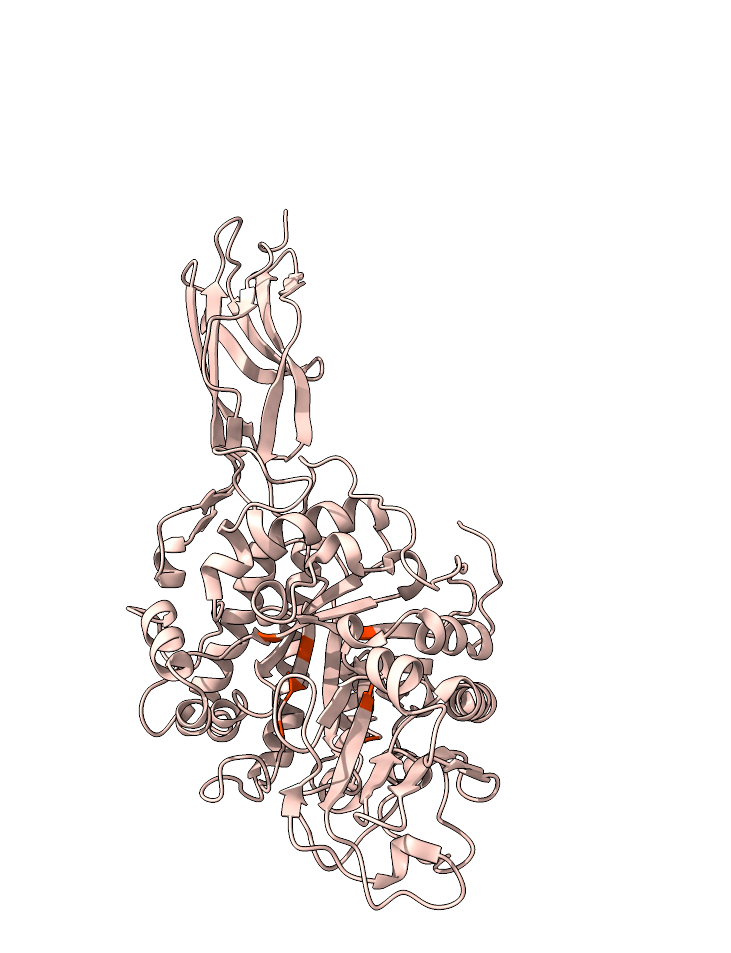


**Supplementary Figure 4.** **Comparison of the structures of GH18 chitinase from *L. braziliensis* (Lbr_ChGH18) and chitinase A from *Serratia marcescens* (SmChitA).** (**A**) Crystal structure of chitinase A from *S. marcescens* (pink) (pdb|1NH6|A Chain A, chitinase A [6]). (**B**) Superimposition of *L. braziliensis* chitinase (blue) onto SmChitA (pink) reveals near-perfect alignment of the domains. Highlighted in red are the catalytic residues whose position in both superposed structures is fully conserved, emphasizing the preservation of the architecture of the catalytic site.


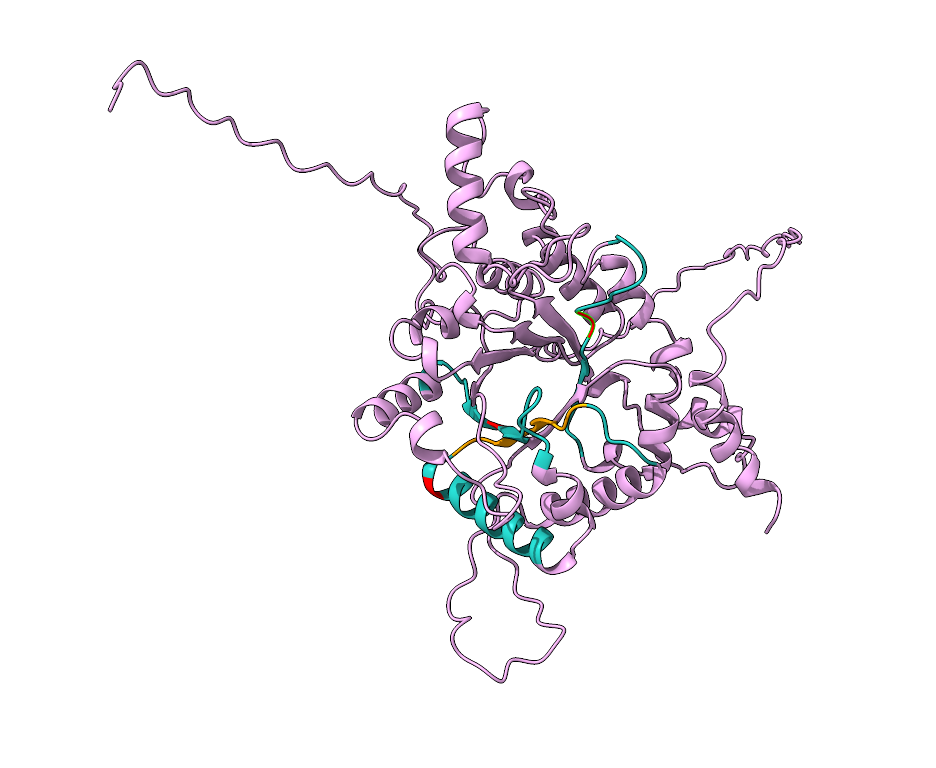

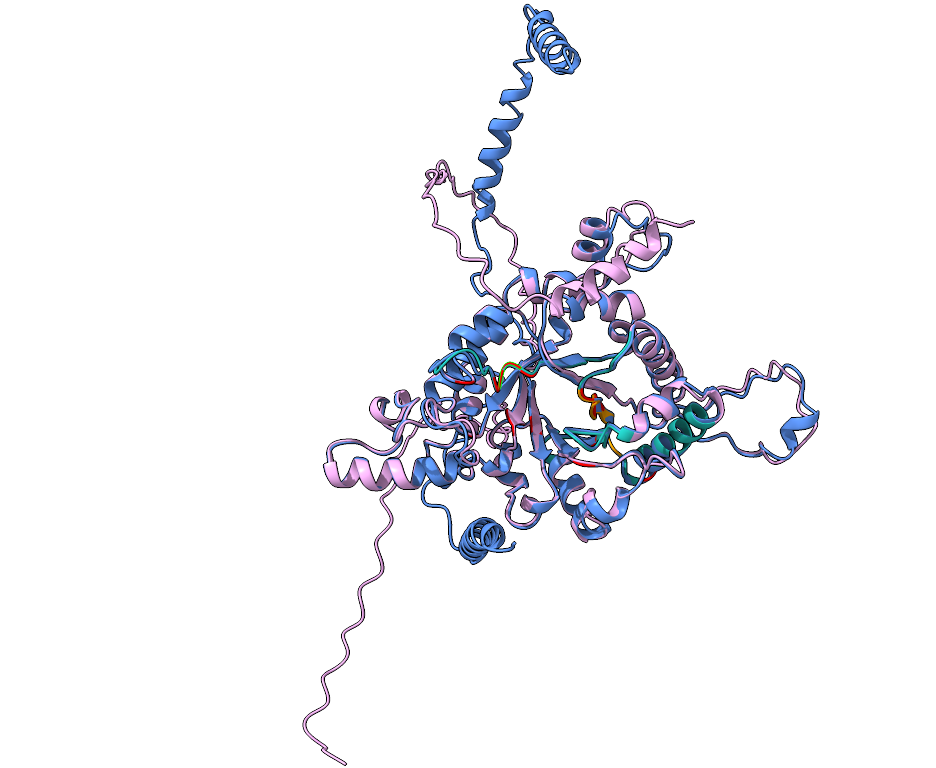

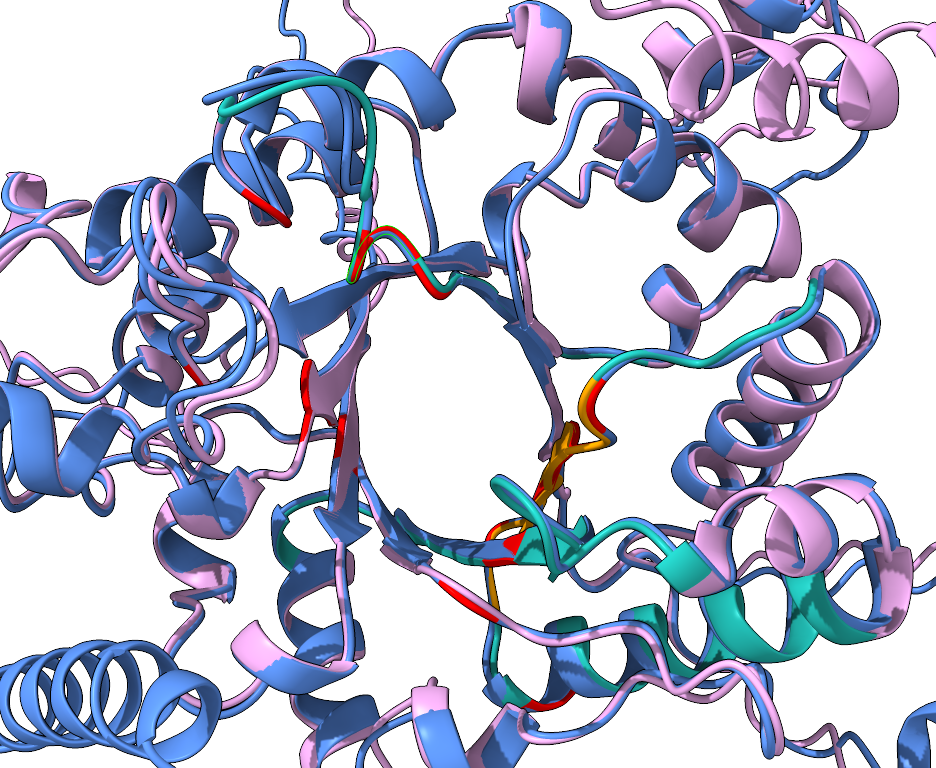


**A**

**B**

**Supplementary Figure 5. Comparative structural analysis of the GH18 chitinases from** ***L. braziliensis* (Lbr_ChGH18) and *L. mexicana* (Lmx_ChiGH18).** (**A**) Predicted three-dimensional structure of **Lmx_ChiGH18** showing the canonical TIM-barrel (β/α)_8_ domain typical of GH18 family enzymes. (**B**) Structural superimposition of Lmx_ChiGH18 (cyan) and Lbr_ChGH18 (blue) reveals an almost perfect overlap of the barrel domain, highlighting the strong conservation of the GH18 catalytic core architecture between both New World *Leishmania* species. Conserved catalytic residues are shown in red and yellow.


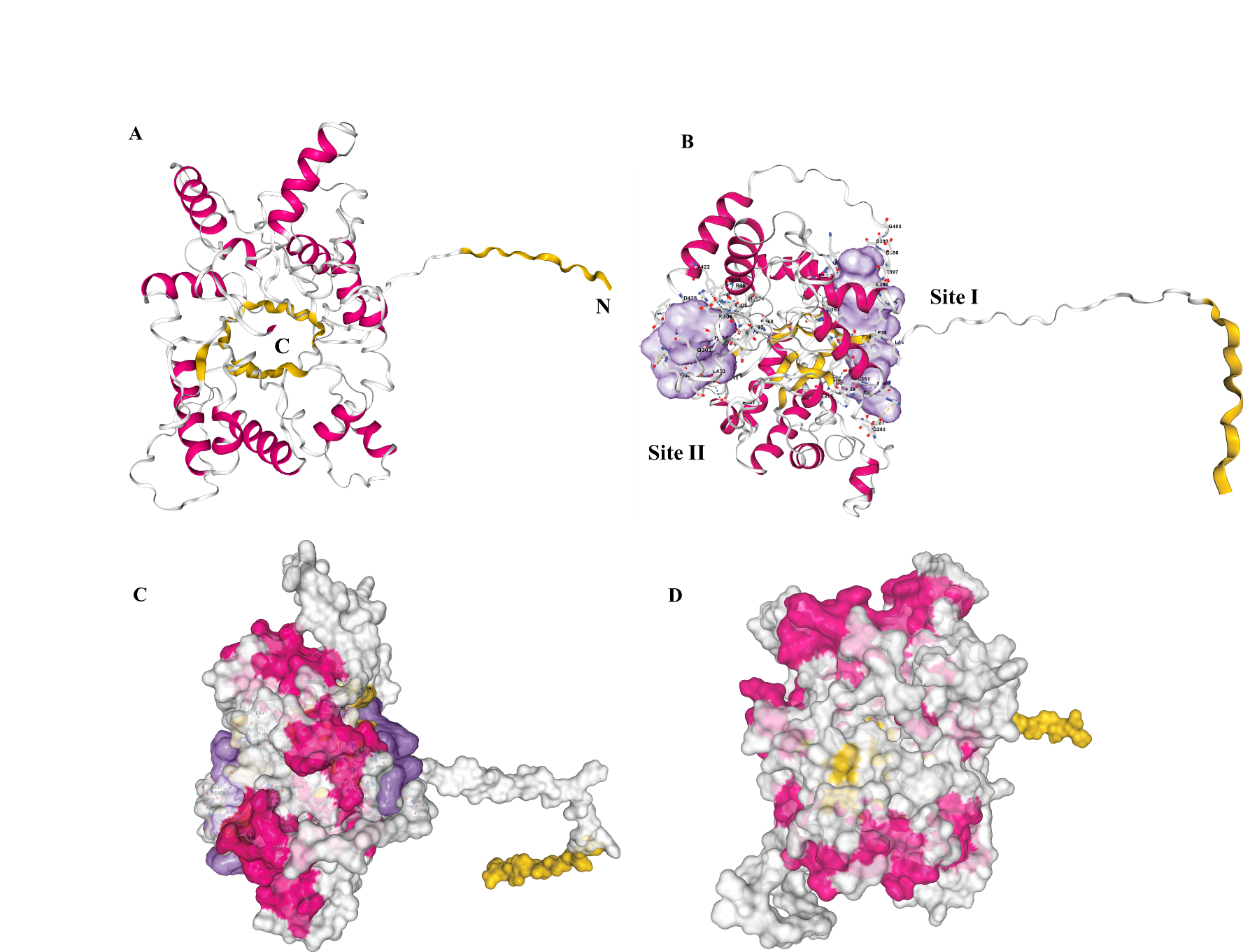


**Supplementary Figure 6. Structural characterization of the processed chitinase of *L. braziliensis* (pLbr_ChGH18)**. (**A)** Three-dimensional structure of the enzyme, highlighting the N- and C-terminal ends. (**B**) Identification of ligand-binding sites I and II, showing GlcNAc₆ bound to the enzyme. (**C**) Surface representation of the enzyme–ligand complex, illustrating the interaction of the GlcNAc_6_ with the chitinase. (**D**) Surface representation of pLbr_ChGH18. Structure coloring: α-helices (dark red), β-strands (yellow), and loops/coils (gray).

**
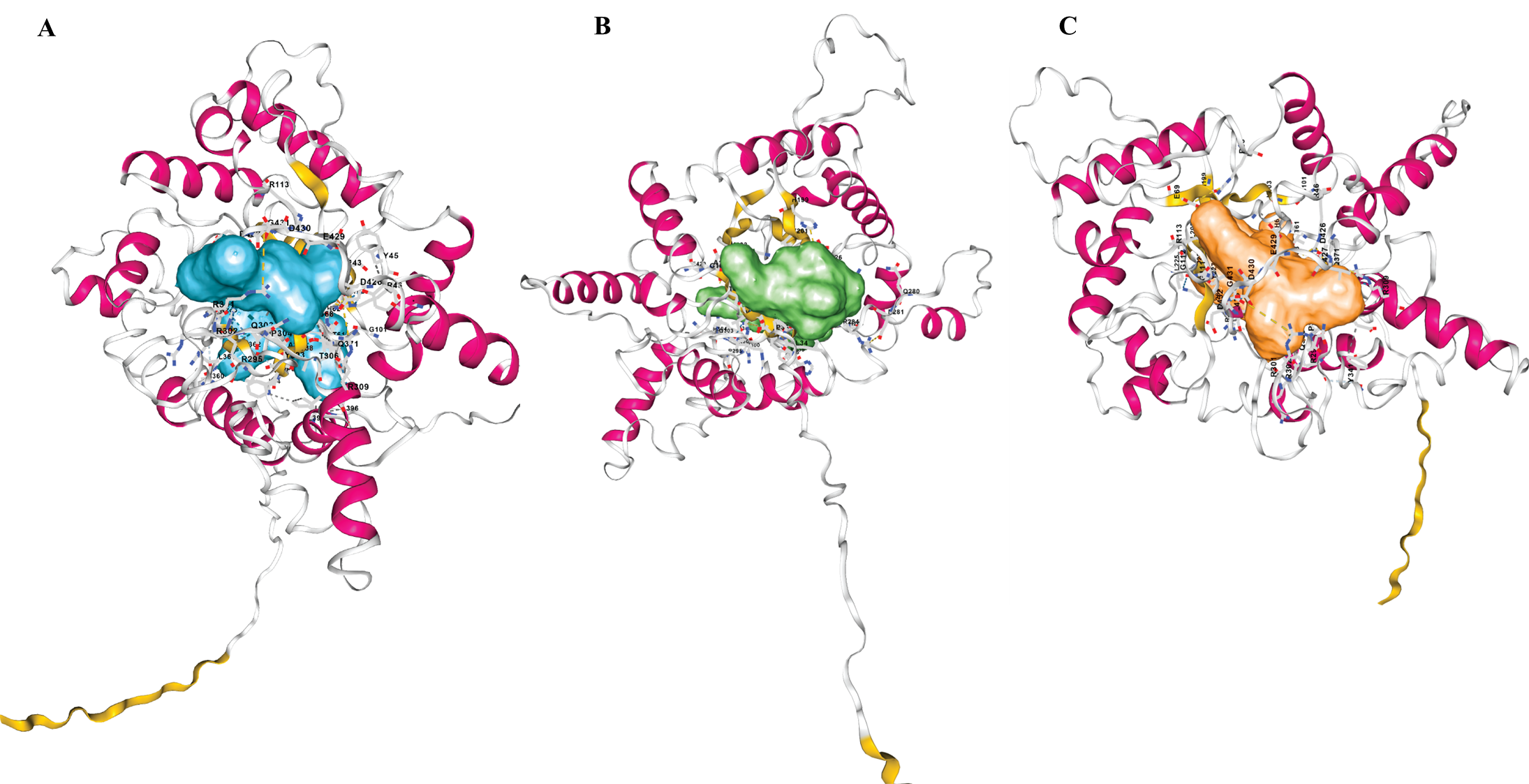
**

**Supplementary Figure 7. The binding sites of ligands on the chitinase of *L. braziliensis* (Lbr_ChGH18) as determined by docking.** Binding sites of closantel (**A**), argifin (**B**), and argadin **(C)** on the chitinase. The ligands are observed binding at both the substrate entry and exit sites of the TIM-barrel, forming a cap-like arrangement that could influence substrate access and release.

**
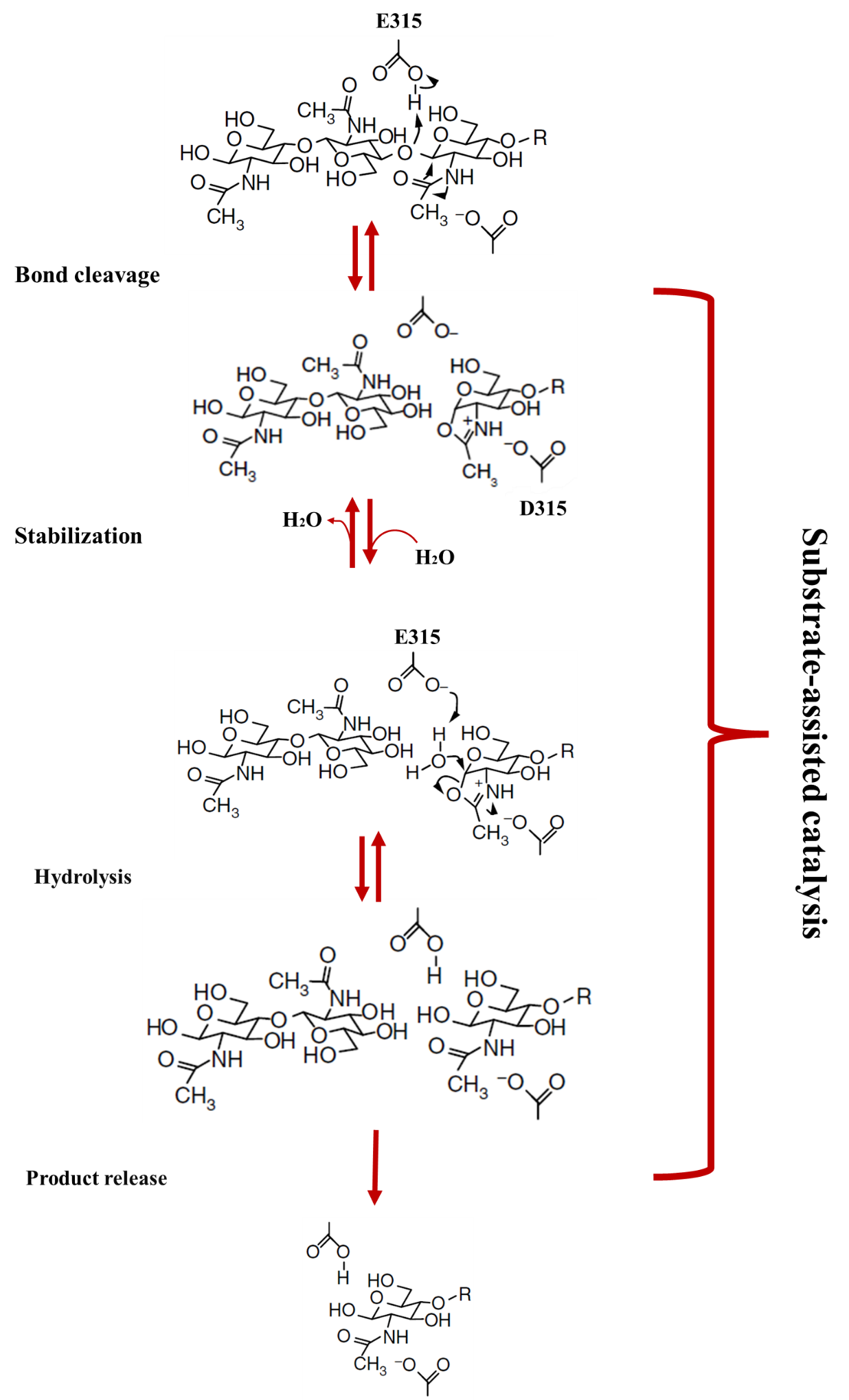
**

**Supplementary Figure 8. Mechanism of glycosidic bond hydrolysis in SmChiA**. Illustrating substrate-assisted catalysis and the roles of key residues in the catalytic process. The four steps shown are: (i) cleavage of the glycosidic bond by the catalytic glutamate residue (E), (ii) stabilization of the oxazolinium ion intermediate by an aspartic acid residue (D), (iii) hydrolysis of the intermediate by a water molecule, and (iv) release of the product. In SmChiA, residue E315 donates a proton to facilitate bond cleavage, while D313 stabilizes the oxazolinium intermediate. Subsequently, E315 abstracts a proton from water, enabling hydrolysis of the intermediate and completing the reaction. Figure based on [7].

**Supplementary Video 1.** Rotating view of the three-dimensional structure of chitinase from L. braziliensis (Lbr_ChGH18), showing secondary elements. Structure coloring: α-helices in purple, β-strands in yellow, and loops/coils in gray.

**Supplementary References**

1. Shanmugasundram, A.; Starns, D.; Böhme, U.; Amos, B.; Wilkinson, P.A.; Harb, O.S.; Warrenfeltz, S.; Kissinger, J.C.; McDowell, M.A.; Roos, D.S.; et al. TriTrypDB: An Integrated Functional Genomics Resource for Kinetoplastida. *PLoS Negl. Trop. Dis.* **2023**, *17*, e0011058, doi:10.1371/journal.pntd.0011058.

2. Ruy, P.D.C.; Monteiro-Teles, N.M.; Miserani Magalhães, R.D.; Freitas-Castro, F.; Dias, L.; Aquino Defina, T.P.; Rosas De Vasconcelos, E.J.; Myler, P.J.; Kaysel Cruz, A. Comparative Transcriptomics in Leishmania Braziliensis: Disclosing Differential Gene Expression of Coding and Putative Noncoding RNAs across Developmental Stages. *RNA Biol.* **2019**, *16*, 639–660, doi:10.1080/15476286.2019.1574161.

3. Shakarian, A.M.; Dwyer, D.M. The *Ld Cht1* Gene Encodes the Secretory Chitinase of the Human Pathogen *Leishmania Donovani*. *Gene* **1998**, *208*, 315–322, doi:10.1016/S0378-1119(98)00011-0.

4. Joshi, M.B.; Rogers, M.E.; Shakarian, A.M.; Yamage, M.; Al-Harthi, S.A.; Bates, P.A.; Dwyer, D.M. Molecular Characterization, Expression, and in Vivo Analysis of LmexCht1: The Chitinase of the Human Pathogen, Leishmania Mexicana. *J. Biol. Chem.* **2005**, *280*, 3847–3861, doi:10.1074/jbc.M412299200.

5. Notredame, C.; Higgins, D.G.; Heringa, J. T-Coffee: A Novel Method for Fast and Accurate Multiple Sequence Alignment1. *J. Mol. Biol.* **2000**, *302*, 205–217, doi:10.1006/jmbi.2000.4042.

6. Berman, H.M.; Westbrook, J.; Feng, Z.; Gilliland, G.; Bhat, T.N.; Weissig, H.; Shindyalov, I.N.; Bourne, P.E. The Protein Data Bank. *Nucleic Acids Res.* **2000**, *28*, 235–242, doi:10.1093/nar/28.1.235.

7. Nakamura, A.; Okazaki, K.-I.; Furuta, T.; Sakurai, M.; Iino, R. Processive Chitinase Is Brownian Monorail Operated by Fast Catalysis after Peeling Rail from Crystalline Chitin. *Nat. Commun.* **2018**, *9*, 3814, doi:10.1038/s41467-018-06362-3.
